# Accumulation Dynamics of Transcripts and Proteins of Cold-Responsive Genes in *Fragaria vesca* Genotypes of Differing Cold Tolerance

**DOI:** 10.3390/ijms22116124

**Published:** 2021-06-07

**Authors:** Isam Fattash, Zachary Deitch, Relindis Njah, Nelson Osuagwu, Vera Mageney, Robert C. Wilson, Jahn Davik, Muath Alsheikh, Stephen Randall

**Affiliations:** 1 Department of Biology and Biotechnology, American University of Madaba, Madaba 11821, Jordan; 2 Biology Department 723 W Michigan Street IUPUI, Indianapolis, IN 46202, USA; zmdeitch@gmail.com (Z.D.); srandal@iupui.edu (S.R.); 3 Department of Biotechnology, INN University, 2318 Hamar, Norway; relindis.njah@inn.no (R.N.); robert.wilson@inn.no (R.C.W.); 4 Department of Clinical Medicine, University of Bergen, Postboks 7804, N-5020 Bergen, Norway; Nelson.Osuagwu@uib.no; 5 Institute for Biology and Environmental Sciences (IBU), Carl von Ossietzky Universität Oldenburg, Carl von Ossietzky-Str. 9-11, 26111 Oldenburg, Germany; v.mageney@gmail.com; 6 Department of Molecular Plant Biology, Norwegian Institute of Bioeconomy Research, Høgskoleveien 8, N-1433 Ås, Norway; jahn.davik@nibio.no; 7 Graminor Breeding Ltd., Hommelstadveien 60, N-2322 Ridabu, Norway; muath.alsheikh@graminor.no; 8 Department of Plant Sciences, Norwegian University of Life Sciences, P.O. Box 5003, 1432 Ås, Norway

**Keywords:** dehydrins, ABA-regulated genes, CBF genes, alcohol dehydrogenase

## Abstract

Identifying and characterizing cold responsive genes in *Fragaria vesca* associated with or responsible for low temperature tolerance is a vital part of strawberry cultivar development. In this study we have investigated the transcript levels of eight genes, two dehydrin genes, three putative ABA-regulated genes, two cold–inducible CBF genes and the alcohol dehydrogenase gene, extracted from leaf and crown tissues of three *F. vesca* genotypes that vary in cold tolerance. Transcript levels of the CBF/DREB1 transcription factor *FvCBF1E* exhibited stronger cold up-regulation in comparison to *FvCBF1B.1* in all genotypes. Transcripts of *FvADH* were highly up-regulated in both crown and leaf tissues from all three genotypes. In the ‘ALTA’ genotype, *FvADH* transcripts were significantly higher in leaf than crown tissues and more than 10 to 20-fold greater than in the less cold-tolerant ‘NCGR1363’ and ‘FDP817’ genotypes. *FvGEM*, containing the conserved ABRE promoter element, transcript was found to be cold-regulated in crowns. Direct comparison of the kinetics of transcript and protein accumulation of dehydrins was scrutinized. In all genotypes and organs, the changes of *XERO2* transcript levels generally preceded protein changes, while levels of COR47 protein accumulation preceded the increases in *COR47* RNA in ‘ALTA’ crowns.

## 1. Introduction

Abiotic stress such as cold is a serious threat to the sustainability of agricultural productivity, causing crop loss worldwide, significantly reducing average yields for most major crops [1]. The diploid woodland strawberry (*Fragaria vesca*) is an herbaceous perennial plant that grows naturally in most regions of the northern hemisphere. *F. vesca* (2n = 2x = 14), is a versatile experimental plant with a small genome (240 Mb). It has a robust genetic transformation system [2] and shares high sequence similarity with the cultivated strawberry (*Fragaria × ananassa*) and other members of the Rosaceae family. *F. vesca* was selected for sequencing as a reference genome for the Rosaceae because of its advantages over other family members, including a short generation time for a perennial, ease of vegetative propagation and small herbaceous stature compared with tree species such as peach or apple [3]. The strawberry plant is highly variable in susceptibility to freezing injury, one of the largest factors affecting crop yield and quality in temperate regions. Winter damage to strawberry plants can result in losses of up to 50% annual yield in production in Scandinavia [4]. Overwintering success relies on the crown to remain unaffected not only by the physical damage induced by freezing but also to survive for extensive periods of time at low sub-zero temperatures, and to be optimally resistant to fungal and bacterial invasion favored by freezing induced damage [5]. Thus, one of the major objectives of a strawberry breeding program is to develop cultivars that are winter hardened and highly cold and freezing tolerant. Identification of molecular markers associated with, or responsible for, low temperature tolerance could be employed in strawberry breeding programs [6]. 

Low temperature (LT) causes oxidative stress, which affects both photosynthetic and respiratory mechanisms in plants. It slows down enzymatic reactions which affect the demand for ATP, leading to an overflow of electrons and an increase in the level of reactive oxygen species (ROS) including superoxide anions, hydroxyl radicals and hydrogen peroxide [7]. Oxidative stress that results from extreme low temperature (freezing) inhibits the activities of the enzymes glutathione reductase, dehydroascorbate reductase and monodehydroascorbate reductase that protect the plants against ROS [8]. Cold stress in plants triggers the activity of a number of genes, which alter the level of metabolites and proteins that are responsible for providing protection against the stress. 

Cold stress signals are transduced through ABA-dependent and ABA-independent pathways [9,10,11]. In addition to the role of the phytohormone abscisic acid (ABA) in various plant developmental processes, such as leaf abscission and initiation of fruit ripening; it is also an important contributor to responses to abiotic stresses. ABA-induced gene expression is mediated by the presence of *cis*-acting elements known as ABA responsive elements (ABREs) [12,13]. The ABA-dependent cold pathway activates genes with ABREs in their promoters, while an ABA-independent (CBF/DREB1) cold pathway activates genes with CRE/DREs in their promoters [14,15]. Transcription factors called the C-repeat binding factors (CBFs) or dehydration-responsive element-binding factors (DREBs) have been identified as major contributors to responses leading to cold/freezing tolerance in *Arabidopsis thaliana* [16] and are indeed essential to develop optimal cold/freezing tolerance [17]. The signaling pathway by which CBFs become activated has been extensively characterized [9,18,19,20]. It is clear that other transcription factors have important contributory roles in the cold response [11,21]. The downstream targets that are coordinately regulated by CBFs include hundreds of genes [22]. Among those are alcohol dehydrogenases (ADH) and dehydrins. Interestingly, these genes can be regulated by both ABREs and CRE/DREs. 

Alcohol dehydrogenase (ADH) is a Zn-binding oxidoreductase that depends on NAD(P)H for interconversion of ethanol and acetaldehyde. ADH is thus associated with alcoholic fermentation where it produces a relatively nontoxic end product of anaerobiotic glycolysis contributing ATP and a small amount of NAD+ that can support glycolysis. Chilling tolerant species are induced towards anaerobic metabolism during LT, which results in increased *ADH* expression [23]. Increases in ADH protein levels also occur in response to cold in both diploid [24] and octoploid species of strawberry [5]. In other species, expression of alcohol dehydrogenase (ADH) is known to increase under various stresses, including low temperature, drought, abscisic acid (ABA) and salinity [25,26,27,28,29,30,31]. In particular, *ADH* genes are among the most commonly found cold-induced genes in cereal crops and Arabidopsis [30]. However, knockout experiments suggest that the single *ADH* gene in Arabidopsis is not required for freezing tolerance [26]. 

Dehydrins (DHNs) belong to a large group of highly hydrophilic proteins known as late embryogenesis abundant (LEA) proteins. A unique feature of all DHNs is a conserved, lysine-rich 15-amino acid domain, EKKGIMDKIKEKLPG [32], the K-segment, which is usually located at the C-terminus with a putative ability to form an amphipathic helix structure that can interact with membrane proteins [33]. Other common features of DHNs are a tract of serine residues (the S-segment), which is part of a conserved motif (LHRSGS4-10(E/D)3) that undergoes phosphorylation and may regulate ion-binding and protein conformation [34,35]. The consensus motif DEYGNP (the Y-segment) found in some dehydrins is located near the N-terminus [36]. DHNs are often classified according to the number and arrangements of these conserved K-, Y- and S- segments [36,37,38]. Evidence supports several proposed physiological functions of dehydrins, including responding to low temperature, drought, salt and heavy metal binding [24,39,40,41,42,43]. In vitro and in vivo studies have shown that some DHNs bind to ions and metals thereby potentially reducing toxicity [44] or the associated production of oxygen free radicals [2,35,45,46]. They may also function as a cryoprotective substance towards freezing sensitive enzymes [47]. While some dehydrin proteins accumulate in seeds late in embryogenesis, distinct dehydrins are present in nearly all the vegetative tissues during normal growth conditions and are highly accumulated in response to abiotic stress [37]. The vegetatively expressed dehydrins in *A. thaliana* are the glutamic acid-rich, acidic DHNs which include COR47, ERD10 and ERD14 and the glycine-rich, neutral/basic DHNs: RAB18, XERO1, and XERO2 [35,37]. Overexpression studies of *DHNs* have shown the positive effects of dehydrin gene expression in enhancing cold tolerance in plants [48,49,50] and particularly in octoploid strawberry [51]. Octoploid strawberry responses to LT include alterations in protein expression and metabolites [5,43,52] which provides enhanced levels of antioxidants, disease resistance proteins and molecular chaperones, as well as protective metabolites such as amino acids, sugars, galactinol and raffinose [43,52]. In *F. vesca* diploids, the protein levels of alcohol dehydrogenase and total dehydrins correlate highly with the degree of tolerance to extensive freezing over a number of distinct genotypes [24]. To examine responses in differentially cold-tolerant plants, diploid strawberry genotypes of *F. vesca* previously characterized as high (‘ALTA’, LT50 = −11.6 °C), moderate/low (‘NCGR1363’, LT50 = −8.2 °C), or low (‘FDP817’, LT50 = −7.7 °C) freezing tolerance [24] were selected for protein and transcript profile analysis. 

We aimed to identify genes potentially contributory to cold tolerance in *F. vesca*, by studying the association of transcript level of homologues of genes known to be involved in freezing tolerance, specifically targeting dehydrins, ABA-responsive genes, and CBFs, and further correlating their expression levels (in the case of dehydrins) with protein abundance. The work described here identifies several of the LT-induced dehydrin proteins in *F. vesca* and compares their expression changes to their encoding transcripts and further extends the evaluation of cold responses to other potential cold responsive genes, characterizing their time-dependent expression, and explores possible mechanisms contributing to cold tolerance.

## 2. Results 

### 2.1. Identification of Putative F. vesca CBFs, ABA-Responsive Genes and Dehydrins 

To identify potential C-repeat binding factors (CBF/DREBs), the NCBI database was searched using BLASTp with the consensus sequence for CBFs (in particular the AP2 domain) as query. Twelve potential Fragaria CBFs (AP2 domain-containing proteins) were identified (Table S1) and aligned with Arabidopsis, tomato and soybean CBF/DREB1s (Figure S1). Two Fragaria CBF candidates (FvCBF1B.1, FvCBF1E) grouped in the same clade as the Arabidopsis, tomato and soybean CBF/DREB1s. CBF1B was the most similar of the remaining candidates and was more distantly related to CBF1B.1. The remaining AP2-containing proteins grouped as DREB2s (drought response element binding proteins) or ERFs (ethylene response factors). A comparison of the diagnostic sequences immediately flanking the AP2 DNA-binding domain [53] showed that the FvCBF1B.1 and FvCBF1E were most similar to the functionally characterized CBFs, while CBF1B.2 lacked similarity immediately downstream of the AP2 domain. Thus only FvCBF1B.1 and FvCBF1E possessed the conserved C-terminal DSAWR consensus sequence. Three putative ABA-responsive genes in *F. vesca* (Table S2) were identified as containing ABREs within 1100 b.p. of their ATGs, (1) GEM-like protein 5-like (XP_004304553.1), (2) BTB/POZ domain-containing protein At5g66560-like (XP_004301873.1) and 3) RING-H2 finger protein ATL11-like (XP_004301345.1). Seven putative *F. vesca* dehydrins were identified (Table S2). Arrangements of the K-, S- and Y-segments in these protein sequences are shown in Figure S2 and sequential relationships and a comparison to Arabidopsis dehydrins is shown in Figure S3. The dehydrins of *A. thaliana* and *F. vesca* cluster in two major phylogenetic groups. Four of the putative *F. vesca* dehydrins (protein FvDehydrin, FvXERO1-like, FvDHN2-like and FvXERO2-like) clustered together in one major group and were closely related to the *A. thaliana* XERO1 and 2, and Rab18 dehydrins. The second major phylogenetic group comprises the COR47 and ERD dehydrins, which includes the two COR47-like (A and B) *F. vesca* proteins. COR47-like A and COR47-like B dehydrins were most clearly distinguished from each other in predicted protein size (179 aa and 246 aa, respectively). The two identified Fragaria dehydrins can be categorized [36,37,38] as SKn (e.g., COR47 A, B) and YnSKn (e.g., XERO2 and CS66-like).

### 2.2. Expression Analysis 

Transcript levels of eight genes (two dehydrin genes, *FvCOR47B* and *FvXERO2* belonging to the LEA protein family group; three putative ABA-regulated genes, *FvGEM, FvBTB* and *FvRHA11*; two cold-inducible CBF genes *FvCBF1B.1* and *FvCBF1E;* and the alcohol dehydrogenase gene *FvADH)* were analyzed in leaf and crown tissues extracted from the three *F. vesca* genotypes (‘ALTA’, ‘NCGR1363’ and ‘FDP817’). Changes in transcript levels were compared across the genotypes to estimate relative responses to cold. When interpreting the impact of fold changes, the absolute starting amount of a transcript present across the genotypes must be considered as that, together with cold-induced changes, contributes to the total amount of transcript abundance. There was little variation in basal levels of most transcripts based upon the Cts at time 0 (Table S3). However, the baseline levels of *GEM*, *CBFs*, *XERO2* and *COR47* transcripts were all consistently higher (lower Ct) in the crowns of the ‘ALTA’ genotype, suggesting elevated levels of transcript in the ‘ALTA’ genotype for these genes under nonacclimating conditions.

### 2.3. Transcript Analysis of FvCBF1B.1 and FvCBF1E 

**Figure 1:**
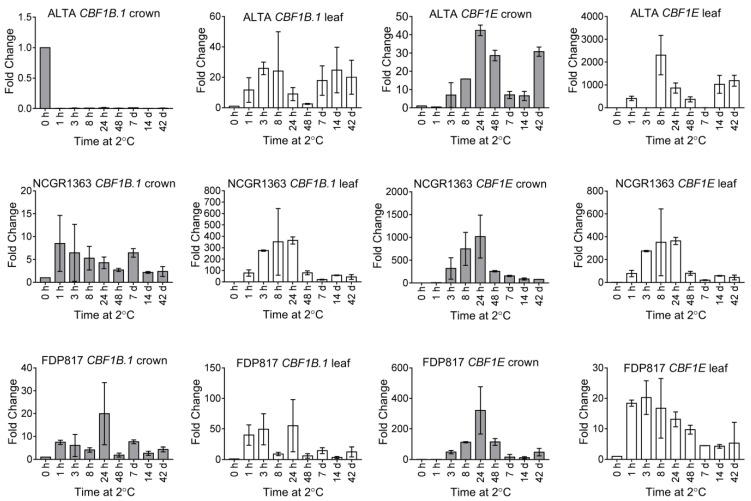
Cold responses of *F. vesca* CBF transcripts. All transcript levels were normalized to *FvPP2A* and expressed relative to untreated control plants (0 h) in response to cold treatment. The crown and leaf tissues from three genotypes ‘ALTA’, ‘NCGR1363’ and ‘FDP817’ were cold treated for 1 h, 3 h, 8 h, 24 h, 48 h, 7 d, 14 d and 42 d, h = hours, d = days. PCR performed as described, with respective primer pairs (Table S4). S.D. are indicated as error bars.

The CBF/DREB1 transcription factors are key to cold responses in most cold-tolerant plants, controlling expression of a regulon that consists of more than 100 genes in Arabidopsis [54]. Two encoded CBFs, which showed the greatest similarity and clustered in phylogenetic analyses with the Arabidopsis, tomato and soybean cold-responsive CBFs, were examined (Figure S1). FvCBF1B.1 was most similar to these orthologs, occupying the same branch as the Arabidopsis, tomato and soybean CBFs. While FvCBF1E had its own branch, it still clustered with the CBF/DREB1s but not with the DREB2s, which are more involved with drought stress responses. Expression analyses showed a modest but rapid up-regulation of *FvCBF1B.1* transcripts in response to cold acclimation (Figure 1). Interestingly, the transcript levels for *FvCBF1B.1* in ‘ALTA’ crowns were strongly decreased after 1 h, persisting throughout the cold exposure. Across the genotypes, the changes in *FvCBF1B.1* transcript levels in crowns were considered quite modest in comparison to other cold responsive plants that showed increases of hundreds- or thousands-fold within 3–6 h [55,56,57,58]. Interestingly, changes in *FvCBF1B.1* transcript abundance tended to be greater in leaf tissues than in the crowns. Alternatively, *FvCBF1E* exhibited strong cold up-regulation, peaking in leaves at 3–8 h, typical of a CBF response pattern (Figure 1). In the crown, a similar pattern was observed but with several hours delay. *FvCBF1E* transcripts exhibited 100-1000-fold increases in leaf tissue in the two more cold-tolerant genotypes (‘ALTA’ and ‘NCGR1363’), which is usual for cold responsive CBFs in both the leaf and crown tissues. 

### 2.4. Transcript Analysis of FvADH 

**Figure 2:**
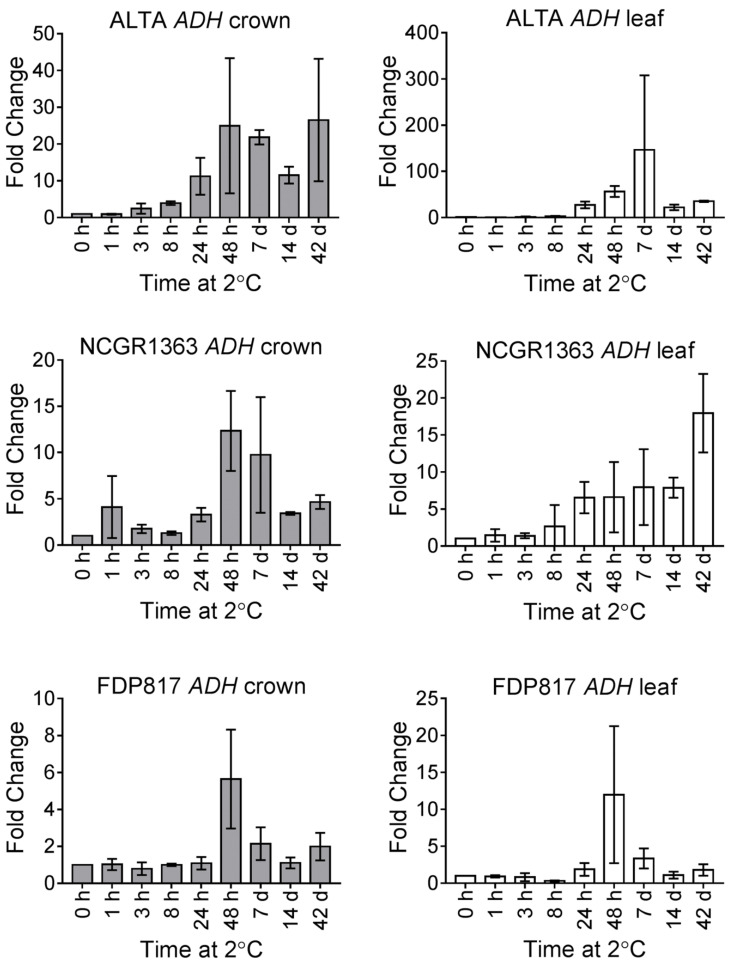
Cold responses of *F. vesca ADH* transcripts. All samples treated as in Figure 1.

Transcript analysis of *FvADH* showed that this gene was up-regulated at 24 h of LT, generally peaking at 48 h after the onset of cold (Figure 2). In crowns of all genotypes, the transcript was most highly expressed at 48 h, and a strong decline in transcript abundance was observed at 7 d and 14 d followed by a slight increase at 42 d of LT. In the ‘ALTA’ genotype, the fold changes were significantly higher in leaf than in crown tissues. It was interesting to note that the changes of *FvADH* transcripts in both crown and leaf in the highly cold-tolerant ‘ALTA’ were much greater than in the other two less cold-tolerant genotypes, ‘NCGR1363’ and ‘FDP817.’

### 2.5. Transcript Analysis of the Dehydrins, FvXERO2 and FvCOR47

**Figure 3:**
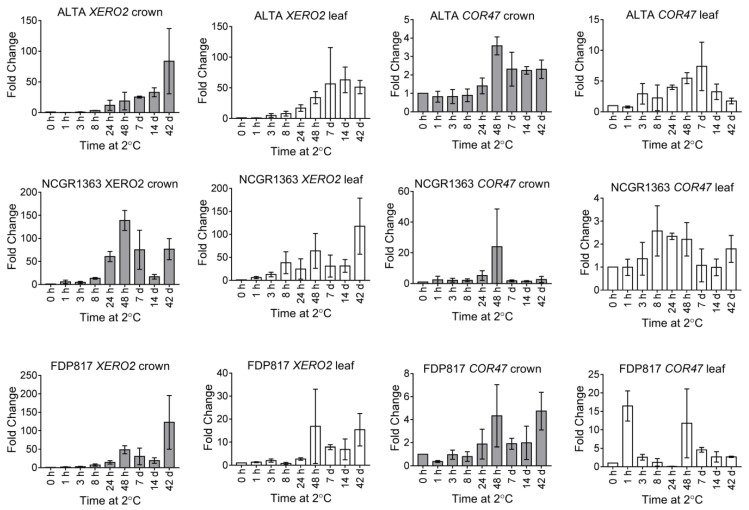
Cold responses of *F. vesca* Dehydrin transcripts. All samples treated as in Figure 1.

*Dehydrin* genes are well documented downstream targets of CBFs. We chose to analyze two homologs of dehydrins shown to be cold responsive in other plants (XERO2 and COR47). The *FvXERO2* mRNA levels were found to increase with similar kinetics in ‘ALTA’ crown and leaf (Figure 3), although transcript increases were initiated slightly earlier in the leaves after the starting of cold treatment. No significant accumulation of the *XERO2* transcripts were observed until 24 h of LT and the highest accumulation levels were generally found in both leaves and crowns at 42 d of LT. Generally, there was a steady and significant increase in transcript levels from 24 h to 42 d of LT, in crown and leaf tissue of all genotypes at 7 and 14 d of LT. A notable exception was a transient decrease observed in both the lesser cold-tolerant genotypes (‘NCGR1363’, ‘FDP817’). The *FvCOR47* transcript was also up-regulated in all cold acclimated plants (Figure 3). In all genotypes, both crown and leaves showed a peak accumulation of *FvCOR47* at 48 h of LT. This transcript showed a lower fold increase across all genotype/tissue-type/time-point-combinations compared to the *FvXERO2* transcripts (approximately 10-fold lower). With the exception of NCGR1363 the changes in *FvCOR47* transcript levels were greater in leaves than in the crown tissues. All genotypes showed similar temporal expression patterns in leaves: a gradual increase in transcript accumulation from 1 h to 48 h, followed by a decrease extending to 42 d of LT. In crowns, though all showed increases in *FvCOR47* transcript levels, no consistent pattern in the transcript levels across genotypes was obvious. 

### 2.6. Transcript Analysis of Putative ABRE-Containing FvGEM, FvBTB and FvRHA11

**Figure 4:**
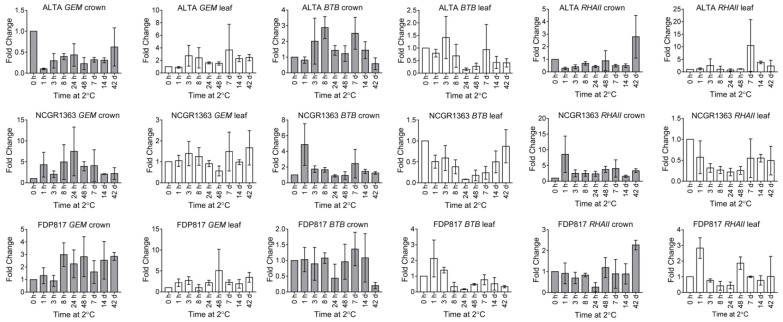
Cold responses of *F. vesca* potential ABA responder transcripts. All samples treated as in Figure 1.

The expression levels of three genes containing putative ABA-responsive elements in their promoters were examined (Figure 4). *AtGEM*-related genes have ABREs, are ABA regulated and are likely functioning as part of the ABA signaling pathway [59]. *FvGEM* transcripts were slightly up-regulated in crowns in response to cold treatment in the two lesser cold-tolerant genotypes with a gradual accumulation over the entire time in the cold (up to five-fold) (Figure 4). The levels of *FvGEM* transcript were decreased in cold-treated ‘ALTA’ genotype crown tissues and increased slightly in the crowns of the other genotypes while no significant differences were observed in the expression in leaves between the three genotypes. BTB/POZ domain containing proteins have been shown to be responsive to ABA, modulating seed germination or ethylene responsive pathways [60]. *FvRHA11* is a member of the ring finger-containing *ATL* (*RHA*) gene family of ubiquitin ligases, some members of which are ABA-responsive [61]. The *ATL* gene family from *A. thaliana* and *Oryza sativa* comprises a large number of putative ubiquitin ligases of the RING-H2 type [61]. LT treatment generally led to strong decreases in *FvBTB* and *FvRHA11* levels in the leaves, maximal at 24 and 48 h. In crowns the expression levels of *FvBTB* and *FvRHA11* were minimal in most genotype/tissue-type combinations (Figure 4). 

### 2.7. Analysis of Total Dehydrin Protein Accumulation 

**Figure 5:**
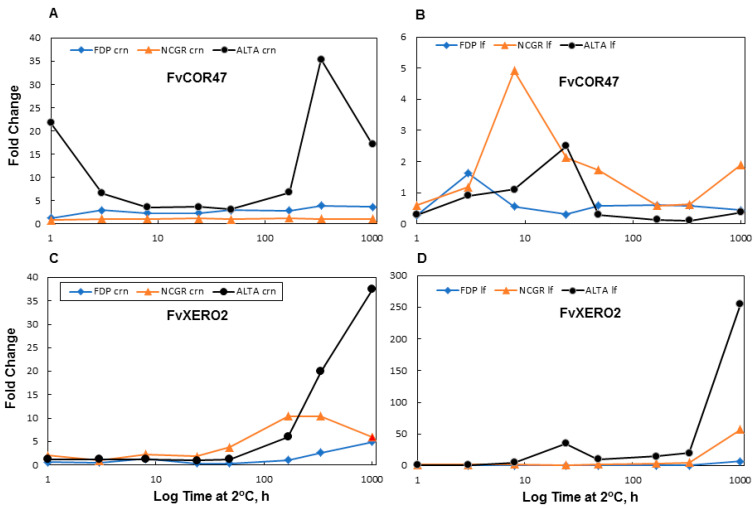
Cold responses of *F. vesca* dehydrin protein. (**A**,**B**) corresponding fold changes of FvCOR47 protein levels in crowns and leaves of three *F. vesca* genotypes in response to cold (2 °C) over time, respectively. (**C**,**D**) are graphs of fold changing of FvXERO2 protein levels in crowns and leaves of three *F. vesca* genotypes in response to cold (2 °C) over time, respectively. All samples were obtained as in Figure 1. Note that although the linear scale on the x-axis is shown in hours, these are the same samples and timepoints as shown in Figure 1, Figure 2, Figure 3 and Figure 4 (i.e., 1008 h time point is 42 d). Genotypes used are designated as FDP indicates the FDP817; NCGR indicates NCGR1363, and ALTA. Crown tissues are indicated as “crn”, leaf tissues as “lf”. Each lane in the gels were loaded with the same total protein (10 μg) and dehydrin levels were determined following Western blotting and were quantitated as described in Methods. Westerns were probed with either anti-COR47 or anti-XERO2 antibodies as indicated.

Having examined the transcriptional response of two *dehydrin* genes (Figure 3) we further examined the general expression pattern of the dehydrin proteins (Figure 5). Dehydrins previously described as 25 kDa, 35–37 kDa and 60–65 kDa (see Supplemental Material in [24]) were detected in *F. vesca* by the antibodies. In general, two patterns of changes in dehydrin protein levels in response to LT were observed. The first pattern observed was a long delay in changes of expression, often no significant increases seen until ca. 7 d (168 h) of LT, after which an increase in dehydrin levels was detected. This was exhibited in the anti-AtXERO2-reactive bands in all genotypes in both crowns and leaves (Figure 5) and for the anti-AtCOR47-reactive bands from FDP817 crowns. The second pattern was an initial rapid response (seen within the first few hours of LT) followed by a decrease and then after 7 d, a strong increase. This was exhibited best by COR47 reactive bands in ‘ALTA’ crowns and less by ‘ALTA’ leaves and ‘NCGR1363’ leaves (Figure 5). ‘NCGR1363’ crowns and ‘FDP817’ leaves showed no significant responses of COR47 reactive proteins to LT.

### 2.8. Identification of COR47-Like and XERO2-Like Proteins and Correlation with Their Respective Rranscripts 

**Figure 6:**
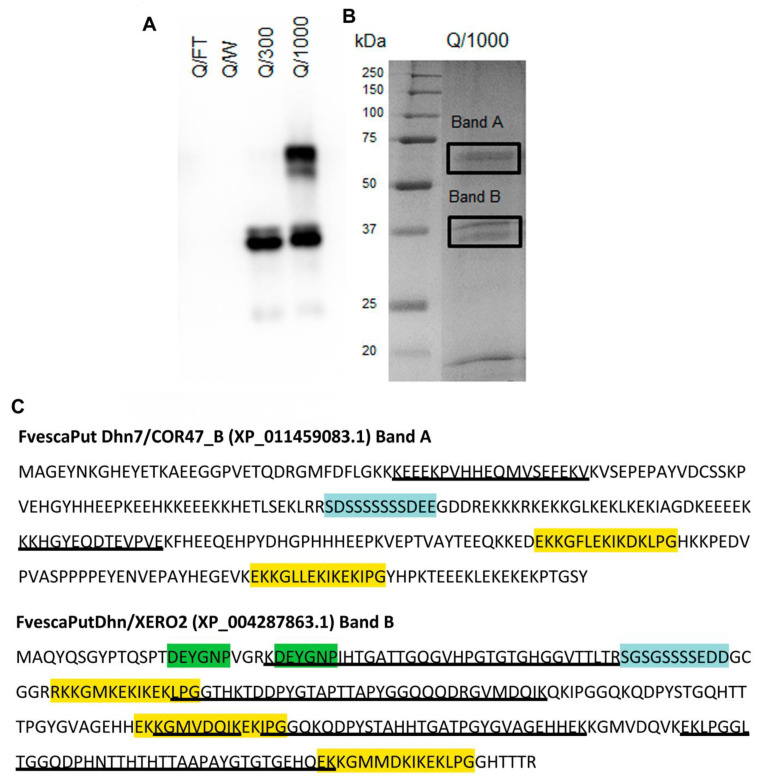
Identification of two dehydrins from *F. vesca*, ‘ALTA’ genotype. (**A**) SDS-PAGE immunoblot of partially purified dehydrins, probed with combination of anti-XERO2 and anti-COR47 antibodies. Q/FT, Q/W, Q/300 and Q/1000 represent flow-through, a 10 mM NaCl wash, a 300mM NaCl elution and a 1000 mM NaCl elution. (**B**) Coomassie-stained SDS-PAGE of the Q1000 fraction (see panel A) indicating with boxed regions, the sectors of the gel removed for mass spectroscopic analysis. (**C**) Amino acid sequence of COR47B with peptides (underlined) obtained by mass spectroscopy (two peptides, coverage = 13%, and the amino acid sequence of XERO2 with peptides (underlined) obtained by mass spectroscopy (five peptides, coverage = 56%).

**Figure 7:**
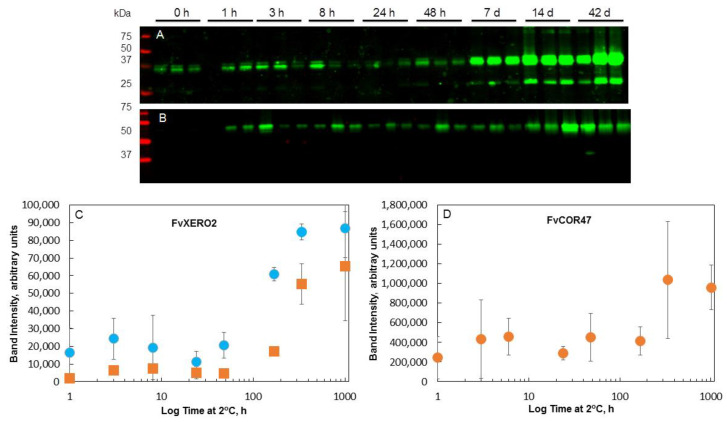
Dehydrin protein accumulation in cold-treated ‘ALTA’ crowns. Panel (**A**), Western blot of genotype ‘ALTA’ probed with anti-XERO2. Panel (**B**), Western blot of genotype ‘ALTA’ probed with anti-COR47. Panel (**C**), quantitation of band intensity in Panel (**A**) (anti-XERO 2). Circles represent the 35-36 kDa band, squares represent the 25 kDa band. Panel (**D**), Quantitation of band intensity in Panel (**B**) (anti-COR47). All signal quantitation are expressed as average (and standard deviation) values of three biological replicates (except for the 1 h time-point which was in duplicate) as shown on gels in panels (**A**,**B**). Replicates acclimated for 0 h, 1 h, 3 h, 8 h, 24 h and 48 h, 7 d, 14 d and 42 d at 2 °C were separated on 12% SDS-PAGE gel, transferred to a nitrocellulose membrane and probed with the respective antibody.

**Figure 8:**
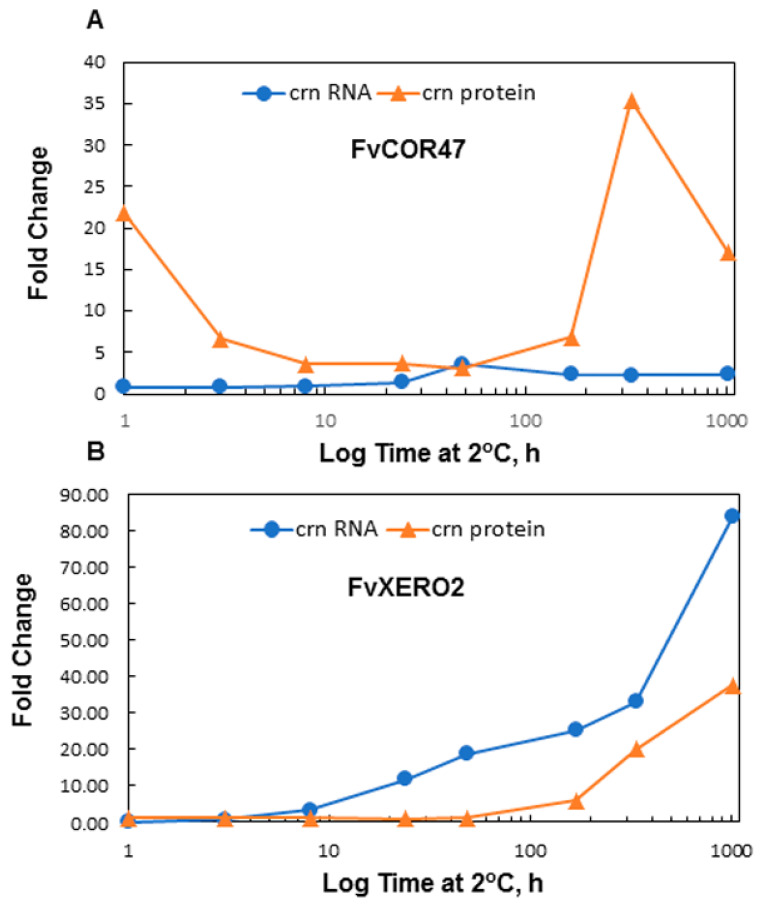
COR47 (**A**) and XERO2 (**B**) transcript and protein levels following LT show distinctive responses in the ‘ALTA’ crown (crn). Relative protein levels (Figure 7) are directly compared to transcript levels (Figure 3).

The accumulation of dehydrin proteins as described above in diploid woodland strawberry is slow relative to the rapid accumulation observed in other cold responsive species [24,43]. The differential expression in the different genotypes (Figure 5) suggested that a closer examination of the kinetics of both transcript and protein accumulation was necessary to understand their possible contribution to the acquisition of cold tolerance in strawberry. To determine the correspondence of specific dehydrin transcripts (qRT-PCR) with their respective dehydrin proteins (Western blots), the identity of the dehydrin proteins was revealed by mass spectroscopy (Figure 6). The dehydrins were purified from ‘ALTA’ crowns (Figure 6A). Two major bands and one minor band corresponded with anti-XERO2 and anti-COR47 activity were eluted in a high salt fraction from an anion exchange column (Q1000), one at 60–65 kDa, a doublet at approximately 37 kDa and a minor band at 25 kDa. The three regions (60–65, 37 and 25 kDa) were excised from the SDS-PAGE gel (the doublet at 37 kDa excised as one) and prepared for analysis by mass spectroscopy. Masses of unique tryptic fragments (underlines in Figure 6C) consistent with distinctive single dehydrin sequences were obtained from the 60–65 (Figure 6A,B) and the 37 kDa (Figure 6B) regions of the gel (Figure 6C). Band 60–65 kDa was thus identified as COR47-like and the 37 kDa band was identified as XERO2-like. Identification of the 25 kDa band was unsuccessful, likely due to the low abundance as indicated by the barely detectable Coomassie-stained band (Figure 6B). The changes in protein bands corresponding to XERO2 and COR47 (Figure 7A–D) were then compared to their respective transcripts (Figure 8). In all genotypes and tissues, the changes in *XERO2* transcript levels generally preceded protein changes (Figure 8 and Figure S5). Similarly, COR47, in the leaves of ‘ALTA’, ‘NCGR1363’ and ‘FDP817’ and in the crowns of ‘NCGR1363’ and ‘FDP817’ the changes in *COR47* transcripts preceded the changes in COR47 proteins (Figure S4). However, in ‘ALTA’ crowns, the most cold-tolerant of all three genotypes, and the genotype showing the greatest increase and greatest absolute levels of COR47 proteins at 42 d, changes in protein were concurrent or even preceded the increases in transcript levels (Figure 8). It is important to mention that the cold-associated changes in *COR47* transcript levels in ‘ALTA’ are lowest of all genotypes, yet the protein levels change the most. We found this quite intriguing that the most cold-tolerant genotype had the poorest correlation between LT-induced abundance changes in COR47 protein and transcripts. 

**Figure 9:**
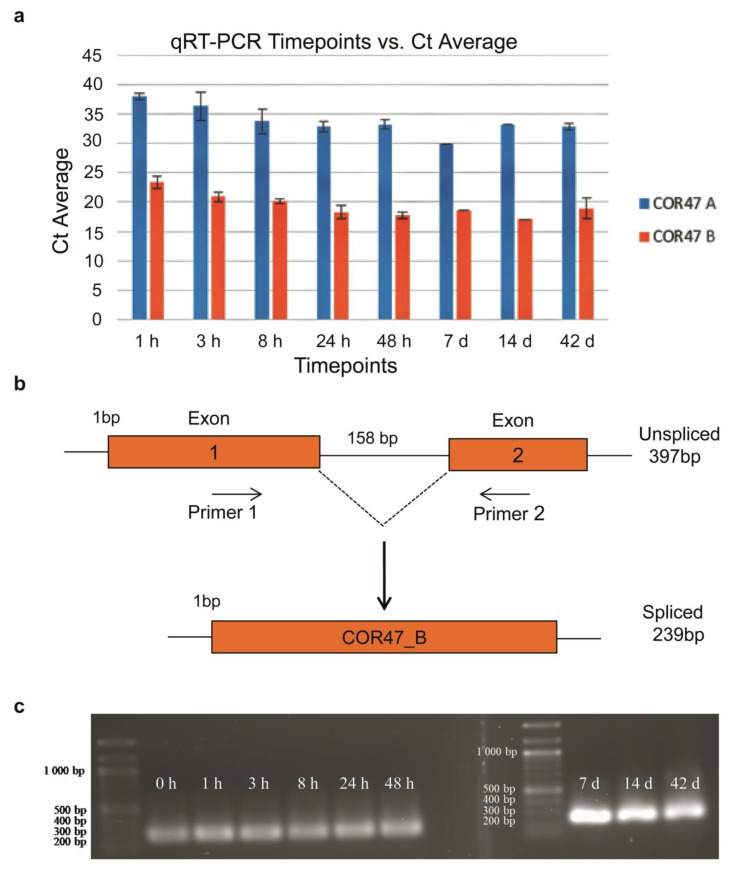
COR47B is the major COR47 transcript and is not alternatively spliced. (**a**) Comparison of transcript levels (Cts) from the distinct *COR47A* and *COR47B* isogenes during cold treatment. Specific primers were developed to distinguish transcripts from the two genes (Table S4). (**b**) Possible RNA products from *COR47B* gene. (**c**) A single spliced COR47B product was seen at all cold time points. Analysis of qRT-PCR products utilizing primers (Figure S1) to amplify the region surrounding the intron of the *COR47B* gene indicates the presence of a single major species consistent with a spliced intron (expected 239 b.p.) compared to an intron-retained product (expected 397 b.p.). All time points of the cold treatment show a similarly sized spliced product. These represent endpoints of qRT-PCR reactions and thus cannot be compared on a quantitative level. Left and right panels were results of samples run on different gels.

To further understand the increase of COR47 protein that anticipates the increase in *COR47* transcript in ‘ALTA,’ we first considered the unlikely possibility that one of the two isogenes of *COR47A* and *B* might be contributing to this interpretation. We considered this unlikely because we only observed a single major antiCOR47 reactive protein band in Western blots (Figure 7B); whereas, if both genes were being expressed, one of them (COR47A) would be predicted to be approximately 50% smaller in mass (see Figure S2). Transcripts derived from *COR47A* and *COR47B* were not distinguished in earlier qRT-PCR experiments (Figure 3), so we developed *COR47A* and *B* transcript specific primers (Table S4) and examined the levels of transcripts in ‘ALTA’ crowns. The *COR47A* gene was expressed at significantly lower levels (>1000 fold lower) than *COR47B*. Thus, *COR47B* seemed likely to be the gene contributing to the majority of the transcript attributed to COR47 (Figure 9a). To address whether an alternative spliced product from *COR47B* (Figure 9b) might explain the changes in COR47-like protein expression, we set out first to examine whether any alternatively spliced products were likely. Since only genomic sequences of *F. vesca* “Hawaii IV” (NCBI) were available, we first sequenced DNA encoding COR47B from all three genotypes used in this study and confirmed that the splice junction sites for the single predicted intron were identical in sequence to that of “Hawaii IV” (data not shown). Using a primer set that flanked the intron, RT-PCR revealed only the fully spliced product, with no evidence for any significant alternatively spliced (or un-spliced) product observed at any time points (Figure 9c). 

## 3. Discussion 

During development and growth, plants endure abiotic stresses which negatively affect crop yields and survival. The diploid Fragaria genotypes (“wild” or woodland strawberries) show significant variation in cold tolerance, with LT50s varying from −8 to −12 °C [24]. Analysis of diploid responses to cold should be useful for understanding more complex genotypes (such as octoploids) for commercial strawberry production.

One important goal of the present work was to identify the likely cold responsive transcripts of CBFs in *F. vesca*. The cold-inducible *CBF* genes are the primary regulators of cold acclimation and the expression of *CBF*s is crucial for freezing tolerance in *A. thaliana* [16,53,62,63]. Increases in *CBF* transcript levels are detectible within 15 min of transferring plants to low temperature. *CBF* transcripts are usually at their highest levels by 4 h of LT at 4 °C, thereafter gradually decreasing to lower levels, though still significantly elevated at 24 h of LT [64,65]. *FaCBF1* (octoploid strawberry) and *PcCBF1* (sour cherry) were found to be up-regulated within 1 h of exposure to cold, peaked at 4 h, then followed a transient decrease in expression level [66]. The two most likely candidates were identified by the presence of critical features including and surrounding the DNA-binding (AP2) sites. In addition to *FvCBF1E’s* very closely related sequence to other characterized CBFs (Figure S1), analysis of the kinetics of cold expression showed a typical CBF cold response. The up-regulation of *FvCBF1E* was highest in the leaves of the most cold-tolerant genotype ‘ALTA’, compared to the two less cold-tolerant genotypes analyzed in this study. Interestingly, in crowns of the ‘ALTA’ genotype, *FvCBF1B*.1 transcripts (the most closely related sequence to Arabidopsis CBFs) were detected with levels much higher than the other genotypes (Table S3) but became undetectable following cold treatment (Figure 1). The *FvCBF1B.1* transcripts were only moderately up-regulated in the crowns of ‘NCGR1363’ and ‘FDP817’ and in the leaves of all genotypes, suggesting that the *FvCBF1B.1* gene product contributes much less to cold tolerance in *F. vesca* tissues. Functional characterization will be necessary to verify the role of *FvCBF1E* in cold tolerance. 

To begin to address the question of whether ABA-responsive genes may contribute to cold-stress tolerance in *F. vesca*, the transcript level of three different *F. vesca* genes containing the conserved ABRE promoter element were investigated during a 42 d LT treatment. Only the *FvGEM* transcript, encoding a protein annotated as a GEM-like protein 5-like (NCBI) and orthologous to the GRAM domain family protein in *A. thaliana* (At5g13200) was found to be regulated in crowns in response to LT treatment (Figure 4). *FvGEM* was strongly down-regulated in ‘ALTA’ crowns and was up-regulated in the two lesser cold-tolerant *F. vesca* genotypes. Transcription of the *A. thaliana* GEM-homolog *(*At5g13200), was up-regulated approximately 10-fold in leaves after 24 h of cold treatment (AtGenExpress Visualization Tool [67,68]). The responses of these genotypes suggest that *FvGEM* may not be suitable as a useful marker for differential LT tolerance in *F. vesca*.

In an earlier study it was suggested that ADH could serve as an important marker for cold tolerance in diploid strawberry [24]. ADH is known to have an important role in other abiotic stress conditions, including hypoxia, and the activity of ADH is vital for plant survival during anaerobic conditions. *ADH* mRNA accumulated to high levels by 48 h in both crown and leaf tissues from all diploid genotypes (Figure 2). The most cold-tolerant diploid strawberry (‘ALTA’) had the greatest fold increase in *ADH* transcripts levels. The strong and sustained increases in *ADH* transcript level described here are consistent with the cold-induced protein levels previously shown (ADH protein levels in *F. vesca* were highly correlated with LT50 (*r* = −0.86, [24]). These results are also consistent with responses in octoploid strawberry (*Fragaria* *× ananassa*) where previous work has shown that during cold-acclimation four ADH protein isoforms accumulated to higher levels in the freezing-tolerant cultivar ‘Jonsok’ compared to the less-tolerant ‘Frida’ and that the protein accumulation peaked after 42 d of LT [5]. As in octoploid strawberry, a key role for elevated levels of ADH contributing to enhanced freezing tolerance is consistent with the ability of ADH to enhance stress survival by alleviating hypoxic conditions caused by ice encasement [69]. 

A typical plant molecular response to cold stress involves expression of members of a family of genes encoding the dehydrin proteins. High accumulation levels of dehydrin proteins in plants are associated with its freezing tolerance [50,51]. Seven putative *F. vesca* dehydrins, showing five distinctive structural types (Figure S2), were identified. These dehydrins cluster in two major phylogenetic groups [24] (Figure S3) similar to those in other plant species [37,70]. In this report, we described changes in abundance of both basic and acidic dehydrin transcripts and, after identifying the proteins by mass spectroscopy, one dehydrin representative from each group: XERO2-like, a basic dehydrin and COR47-like, an acidic dehydrin were chosen. *FvXERO2* transcripts were highly up-regulated in both ‘‘ALTA’’ crown and leaf tissues from 48 h to 42 d of LT, peaking at 42 d. The most cold-tolerant genotype, ‘ALTA’, accumulated the highest *FvXERO2* transcript levels compared to the two less cold-tolerant genotypes ‘NCGR1363’ and ‘FDP817’, similar to results found previously in octoploids [5]. *FvXERO2* transcripts, thus, showed strong and consistent increase over the entire exposure to cold. Protein levels also increased, with a slight delay (relative to the *XERO2* transcripts), consistent with *XERO2* transcripts driving increases in XERO2 protein accumulation. 

A modest increase in transcript levels of a representative of the acidic dehydrins, *Fv*COR47, peaked at 48 h followed by a decreased level in accumulation until 42 d. This is similar to the pattern observed in *A. thaliana*, where *COR47* has been shown to be transiently up-regulated during LT and then decreases in expression after 14–21 d of LT [71]. However, it is quite different from the pattern found in octoploid cold-tolerant ‘Jonsok’ where a rapid, but transient cold response accumulation was observed, peaking at 1 d of LT [5]. 

In the ‘ALTA’ genotype, decreases in transcript levels were incongruous with the increases in COR47 protein levels (Figure 8). Possible explanations, such as the alteration of levels of COR47 isoforms or alternative splicing, were explored. The possibility of the *COR47A* gene contributing significantly at the protein level seemed quite unlikely, as its transcript levels were several orders of magnitude lower than the major isoform. Further, the predicted *COR47A* protein was smaller and was expected to be readily resolved from the COR47B protein and thus was not included in the COR47B protein quantitation. In terms of alternative splicing, the *COR47B* gene has only a single predicted intron and no unspliced products and no alternatively spliced products were observed at any LT treatment time point. The mechanism for the post-transcriptional regulation of COR47 must be further explored. 

## 4. Materials and Methods 

### 4.1. Plant Material 

Plant runners of three *F. vesca* genotypes (‘ALTA’, ‘FDP817’ and ‘NCGR1363’) were planted in a peat-based potting compost (90% peat, 10% clay) containing 1:5 (v/v) granulated perlite in a 10 cm plastic container. The plants were flow-irrigated twice a week with a solution containing 7.8 mM nitrogen, 1 mM phosphorus and 4.6 mM potassium per liter. The plantlets were propagated in a greenhouse with supplementary light (temperature 18 ± 2 °C and 20 h photoperiod) and grown for 42 d. Cold acclimation (CA) was performed by transferring plants to a cold room at 2 °C with a short-day photo-period (Supplemental light was provided by high-pressure sodium lamps (SON-T), 10 h light/14 h dark at 90 µmol m^−2^ s^−1^). Whole plant leaves and entire crown tissues were harvested and flash frozen in liquid nitrogen after 0 h (untreated), 1 h, 2 h, 3 h, 8 h, 24 h, 48 h, 7 d, 14 d, and 42 d of low temperature treatment. The 0 h untreated control plants were harvested at room temperature while the cold treated plants were harvested in the cold room. Triplicate samples (biological replicates, each replicate containing material from 5 plants) of both leaves and crown tissues from each genotype were harvested for each time point and stored at −80 °C until RNA or protein extraction. 

### 4.2. Identification of Putative F. vesca ABA-Responsive and Cold-Responsive Genes

Potential ABA and cold responsive genes in *A. thaliana* were identified by BLASTn searches using the ABRE promoter consensus sequence motif (ACGTGGC/T) as a query against the RefSeq *A. thaliana* database. Selected genes that contained ABREs and that were functionally annotated as cold responsive in Arabidopsis (TAIR; www.arabidopsis.org) were used as query sequences in a second BLASTn search to identify homologous sequences in the *F. vesca* reference genome (http://www.phytozome.net (accessed on December 2014)). All blast searches were performed using default parameters. Putative *F. vesca* dehydrin proteins were identified by performing BLASTp searches using the K-segment motif (EKKGIMDKIKEKLPG), which is considered diagnostic for dehydrins, as query against the *F. vesca* RefSeq Protein Database at NCBI. The blast search parameters were adjusted for small input query sequences. These full-length sequences were then queried against the latest annotation of *F. vesca* v.4.a2 (ftp://ftp.bioinfo.wsu.edu/species/Fragaria_vesca/Fvesca-genome.v4.0.a2/ (accessed on 10 September 2020)) for updated gene IDs and BLASTp-based homologies of predicted protein sequences against the plant NR protein database (ftp://ftp.bioinfo.wsu.edu/species/Fragaria_vesca/Fvesca-genome.v4.0.a2/homology/blastp_Fvesca_v4.0.a2_vs_nr.xlsx.gz (accessed on 10 September 2020)). Identified sequences were imported to CLC Genomic Workbench (Qiagen, Hilden, Germany) and annotated for the presence of conserved dehydrin sequence motifs, including the Ser-cluster, Y-segment and the K-segment. This was done by creating a local database consisting of the putative *F. vesca* sequences and searching it with the Y- and K-segment consensus sequences as queries, with a manual supervised curation by one of the authors. A phylogenetic analysis was performed using Clustal Omega to build the multiple sequence alignment, and then a phylogenetic tree was constructed using NJPLOT using the neighbor joining method [72].

### 4.3. qPCR and Verification of Amplification Products 

RNA was extracted from 100 mg *F. vesca* crown and leaf tissues using Spectrum Plant Total RNA Kit (Sigma Aldrich, St. Louis, MO, USA) according to the manufacturer’s instructions. Prior to reverse transcription, traces of residual genomic DNA were removed by digestion of 2 µg RNA with 1 U RNase-free amplification grade DNase I (Invitrogen, Carslbad, CA, USA) in a total reaction volume of 22 µL in accordance with the manufacturer’s instructions. Of 36 representative RNA samples tested, 19 had RIN values of 9–10, 16 had 8–9 and one had a RIN value of 7.8. Gene-specific primers for qPCR were designed for 10 targets (Table S4). Transcript *FvPP2A* (serine/threonine protein phosphatase 2A), known to be stable to cold treatment in Arabidopsis [73] and verified to be stable in response to cold treatment based on RNASEQ data in both *F*. *ananassa* and *F*. *vesca* (data not shown), was used for qPCR data normalization. Primers were designed using CLC Genomics Workbench 11 (Qiagen, Hilden, Germany) using the following parameters: primer length 17–26 nt; melting temperature (Tm) 59–62 °C; %GC content 30–70; NaCl 50 nM; dNTPs 0.1 mM and MgCl_2_ 1.5 mM. The qPCR amplicon lengths were between 90–180 base pairs. First strand cDNA synthesis was performed with 1 µg DNase I-treated total RNA using 200 U Superscript TM III reverse transcriptase (Invitrogen, Carslbad, CA, USA), primed with 2.5 µM random hexamer primers (pdN6) and 250 ng oligo (dT)18 primers, in a total reaction volume of 20 µL according to manufacturer’s instructions. Transcript levels of each target were analyzed by real time qPCR using EvaGreen^®^ to monitor dsDNA synthesis. Each reaction was composed of 2 µL 5X Hot FIREpol EvaGreen qPCR Mix plus ROX (Solis BioDyne, Tartu, Estonia) and 0.1 µM gene-specific primers in a total reaction volume of 10 µL. qPCR was performed with the AB17500 real time PCR detection system (Applied Biosystems, Foster City, CA, USA) using the following thermal cycling parameters: 50 °C for 2 min, 95 °C for 12 min, then 40 cycles of 95 °C for 30 s, 60 °C for 1 min, 72 °C for 1 min, and a final dissociation step. ROX was used as the internal passive reference dye to normalize the fluorescent reporter signal. Post-qPCR dissociations were performed to verify a single peak and at least one amplicon from each primer set was sequenced to verify the appropriate target. PCR efficiencies (80–100%) were determined by a linear regression method (utilizing the LinRegPCR program; [74]), and the average PCR efficiency values for each respective amplicon group were utilized. The expression level of each transcript was normalized to the reference gene *FvPP2A* used as internal loading control. Relative transcript levels compared to the control sample (0 h untreated plant) were calculated using the Pfaffl method [75]. 

### 4.4. Western Blotting 

Total protein was extracted from the same experimental plant material as that used for the transcript analyses to investigate dehydrin protein accumulation. Powders from crown and leaf tissue (50 mg) were homogenized in hot 2x SDS-PAGE sample buffer (SSB) containing 100 mM DL-dithiothreitol (DTT), 1.5 M Tris-HCl (pH 8.5), 2% glycerol, 2% SDS (*w*/*v*), 2% mercaptoethanol and 1x protease inhibitor cocktail (Roche Complete TM protease inhibitor cocktail; Roche, USA). Protein concentrations were estimated using the Amino Black method [76]. Three biological replicates (each of which were composed of 5 individual plants) obtained from each time-point and from all three genotypes were analyzed by Western blotting with a primary antibody raised against the K-sequence motif, which is diagnostic of dehydrin proteins in plants [36,37,38], anti-AtXERO2 or anti-AtCOR47 primary antibodies as described in [35]. Secondary antibody was anti-rabbit IgG (1:8000, Alexa Fluor 790). After appropriate washing, an image was taken using a Li-Cor Odyssey CLx Imager (169 um resolution) and quantitated using Image Studio Lite Ver 5.2. The relationship between fluorescence vs protein levels were linear up to approximately 10 μg total protein extract (Figure S5). Protein loads for all Western blot analysis were kept at or below 10 μg.

### 4.5. Dehydrin Extraction, Fractionation and Identification by Mass Spectrometry 

Tissue from 42 d cold treated ‘ALTA’ crown tissues (1.5 g fresh weight) was extracted in a denaturing buffer, as described for Western blots. This rigorous extraction method was necessary for quantitative removal of dehydrins from crown tissues. The homogenates were then centrifuged for 10 min at 10,000× *g* to remove debris and 5 volumes of 0.1 M Ammonium Acetate in 100% Methanol were added to the supernatants. The supernatants were stored overnight at −20 °C, then centrifuged for 10 min at 10,000× *g*. The pellets were resuspended in 0.2% (*w*/*w*) Triton X-100 and heat-treated in an 80 °C water bath for 10 min. The heat-treated samples were then centrifuged for 10 min at 10,000× *g* and supernatants were stored at −80 °C. The Triton X-100 supernatant was diluted 10-fold with 10 mM Tris-HCl buffer, pH 8.5 (at 4 °C), and loaded at 0.5 mL.min^−1^ onto a 50 mL packed bed volume of DEAE-Sepharose (Amersham-Pharmacia Biotech, Uppsala) anion-exchange column. The proteins were eluted with a linear 10 to 300 mM NaCl gradient, followed by a 1 M NaCl elution generated by a Waters 650E Advanced Protein Purification System (Millipore, Bedford, MA, USA). Fractionated protein samples were separated by 12% SDS-PAGE gels as the final purification step. Western blots were performed and Coomassie-stained dehydrin protein bands corresponding to apparent mass of 35–37 kDa (later identified as XERO2; XP_004287863.1), 65–70 kDa (later identified as COR47; XP_011459083.1), or 25 kDa (unsuccessfully identified; note region was excised even though no visible Coomassie-stainable band was present) from duplicate gels were excised with a sterile blade. The gel bands were destained, reduced with 10 mM DTT in 10 mM ammonium bicarbonate and then alkylated with 55 mM iodoacetamide (prepared in 10 mM ammonium bicarbonate). Alkylated samples were digested by trypsin (Promega) overnight at 37 °C. Digested peptides were sequentially extracted from the gel spots with: (1) 50% ACN/49.9% H_2_O/0.1% TFA; and then (2) 99.9% ACN/0.1% TFA. The combined extracts of tryptic peptides were injected into a C18 column (TSKgel ODS-100 V, 3 μm, 1.0 mm × 50 mm). Peptides were eluted with a linear gradient from 5 to 35% acetonitrile (in water with 0.1% FA) developed over 60 min at room temperature, at a flow rate of 50 uL.min^−1^, and the effluent was electro-sprayed into an LTQ mass spectrometer (located at the Indiana University Purdue University, Indianapolis Proteomic Core Facility). The acquired data were searched against NCBI protein sequence database of *F. vesca* (http://www.ncbi.nlm.nih.gov/, 57918 entries (accessed on 11 July 2016)) using Sequest^TM^ algorithms. 

## 5. Conclusions

The cold responsive CBF has been tentatively identified as FvCBF1E (XP_004298771.1). Interestingly, the similarity of changes of cold-induced *CBF* levels in the highly cold-tolerant and less tolerant genotypes suggest that the differential cold sensitivity amongst the genotypes may be due to CBF-independent events. The examination of the various transcripts reveals a complexity of both temporal and genotype responses to cold. The changes in transcript levels shown here, together with protein changes [24] are consistent with an important role of ADH in cold tolerance. Dehydrins likely also play an important role in cold acclimation and protein levels correlate well with cold tolerance among the genotypes. In the case of COR47B, it appears that post-translational regulation is important for control of dehydrin protein levels. While requiring further investigation, these findings suggest that post-transcriptional cold-regulation of protein levels may be an important contributor to cold tolerance mechanisms. 

## Data Availability

Data is contained within the article or Supplementary Material.

## Supplementary Materials

The following are available online at https://www.mdpi.com/article/10.3390/ijms22116124/s1, Figure S1: Phylogenetic analysis showing the relationship between *F. vesca (Fv)* putative CBFs (AP2 containing) and *A. thaliana (At), S. lycopersicum (Sl),* and *G. max (Gm)* CBF/DREBs. Figure S2: Annotation of *F. vesca* dehydrin proteins and their Y-, K-, and S-segments. Figure S3: Phylogenetic analysis showing the relationship between ten *A. thaliana* dehydrins and the seven putative *F. vesca* dehydrin proteins identified in this study. Figure S4: Corresponding changes of FvXERO2 and FvCOR47 transcript and protein in leaves and crowns of three *F. vesca* genotypes in response to LT. Figure S5: Quantitation of FvCOR47 is linear over a 10-fold range of protein (maximum of 10 μg). Table S1: (A) Alignment of the CBF candidates; their AP2 domain and immediately flanking regions are compared to the CBF consensus (JAGLO). (B) Amino acid sequences used for phylogenetic analysis of CBFs. (C) Amino acid sequences used for phylogenetic analysis of Dehydrins. Table S2: *Fragaria vesca* and other genes relevant to this study. Table S3: Summary of threshold Cts for all targets under non-acclimating conditions (0 h in the cold). Table S4: qRT-PCR primers used in this study.
