# Supplementary material for: Accumulation Dynamics of Transcripts and Proteins of Cold-Responsive Genes in Fragaria vesca Genotypes of Differing Cold Tolerance
Source: Int J Mol Sci. 2021 Jun 7;22(11):6124. doi: 10.3390/ijms22116124 (PMC8201005; doi:10.3390/ijms22116124)
Supplement: Supplementary file 1 [file ijms-22-06124-s001.zip › Supplementary Materials/Fattash et. al. Supplementary Materials.pdf]

Accumulation dynamics of transcripts and proteins of cold-responsive genes in *Fragaria vesca* genotypes of differing cold tolerance

Isam Fattash [1,*], Zachary Deitch[2], Relindis Njah[3], Nelson Osuagwu[4], Vera Mageney[5], Robert C. Wilson[3], Jahn Davik[6], Muath Alsheikh[7,8] and Stephen Randall[2]

1 Department of Biology and Biotechnology, American University of Madaba, Madaba, Jordan; i.fattash@aum.edu.jo (I.F.)
2 Biology Department 723 W Michigan Street IUPUI, IN, USA; zmdeitch@gmail.com (Z.D.); srandal@iupui.edu (S.R.)
3 Department of Biotechnology, INN University, Hamar, Norway; relindis.njah@inn.no (R.N.); robert.wilson@inn.no (R.C.W.)
4 Department of Clinical Medicine, University of Bergen, Postboks 7804, N-5020 Bergen, Norway; Nelson.Osuagwu@uib.no (N.O.)
5 Institute for Biology and Environmental Sciences (IBU), Carl von Ossietzky Universität Oldenburg, Carl von Ossietzky-Str. 9-11, 26111 Oldenburg, Germany; v.mageney@gmail.com (V.M.)
6 Department of Molecular Plant Biology, Norwegian Institute of Bioeconomy Research, Høgskoleveien 8, N-1433 Ås, Norway; jahn.davik@nibio.no (J.D.)
7 Graminor Breeding Ltd., Hommelstadveien 60, N-2322 Ridabu, Norway Department of Plant Sciences, Norwegian University of Life Sciences, P.O. Box 5003, 1432 Ås, Norway; muath.alsheikh@graminor.no (M.A.)
8 Department of Plant Sciences, Norwegian University of Life Sciences, P.O. Box 5003, 1432 Ås, Norway; muath.alsheikh@graminor.no (M.A.)
* Correspondence: i.fattash@aum.edu.jo (I.F.)

- **SUPPLEMENTARY FIGURES 1-5 AND SUPPLEMENTARY TABLES 1, 2 and 4**

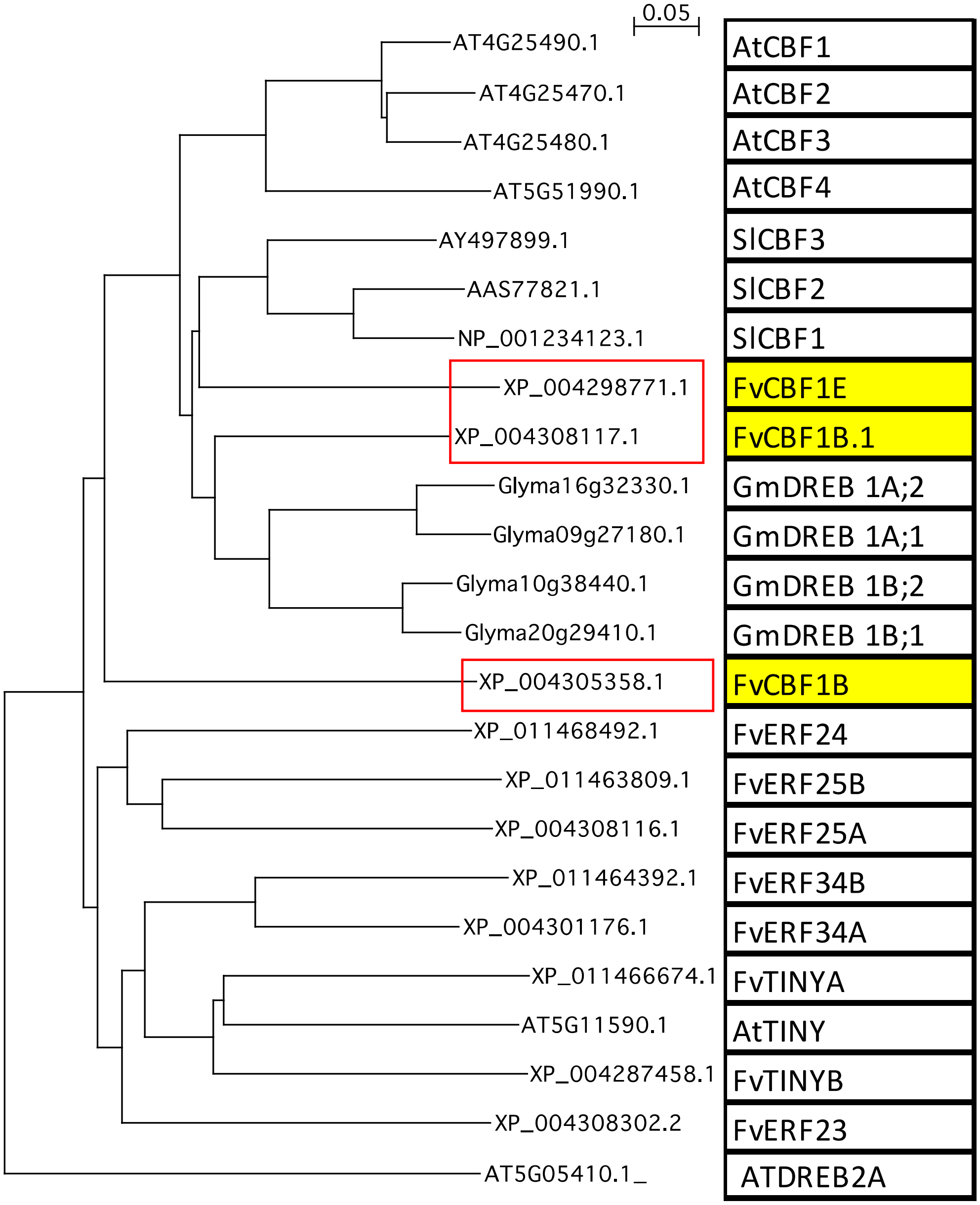

Figure S1: Phylogenetic analysis showing the relationship between *F. vesca (Fv)* putative CBFs (AP2 containing) and *A. thaliana (At)*, *S. lycopersicum (Sl)*, and *G. max (Gm)* CBF/DREBs. To evaluate possible candidates for a *F. vesca* cold responsive CBF, all *F. vesca* AP2 DNA-binding domain containing proteins were compared with selected Arabidopsis, tomato, and soybean CBFs. Shaded boxes (yellow, red) are those *F. vesca* CBFs considered best candidates for CBFs. ATDREB2A was included as an outlier. See Table S1A for visualization and alignment of the AP2 domain and flanking regions and Table S1B for protein sequences utilized.

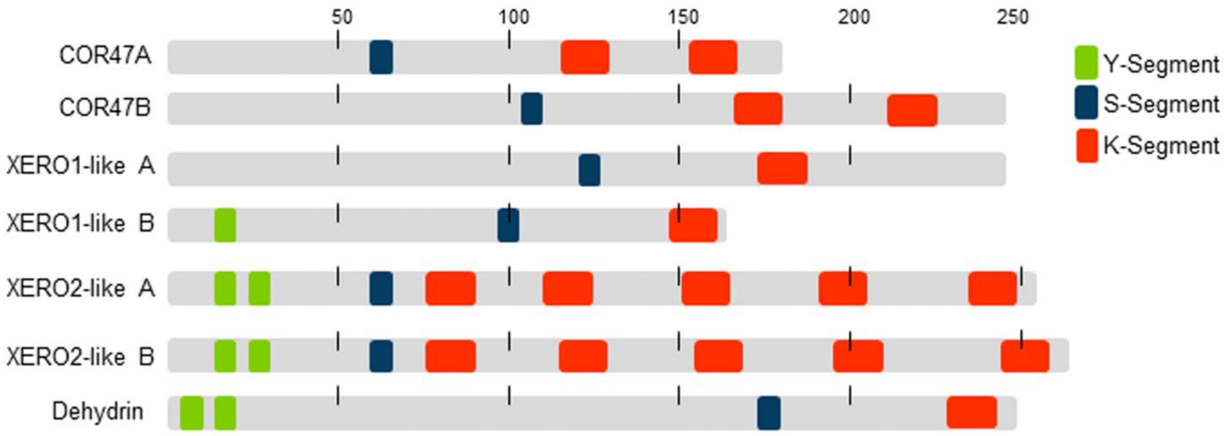

Figure S2: Annotation of *F. vesca* dehydrin proteins and their Y-, K-, and S-segments. Putative dehydrins were identified in BLASTp searches using the conserved consensus sequences EKKGIMDKIKEKLPG (K) and DEYGNP (Y) as queries in a local database containing the putative *F. vesca* dehydrins. Note that XERO2-like A and B are alternative transcripts derived from the same gene. The two predicted protein products differ slightly in sequence and mass.

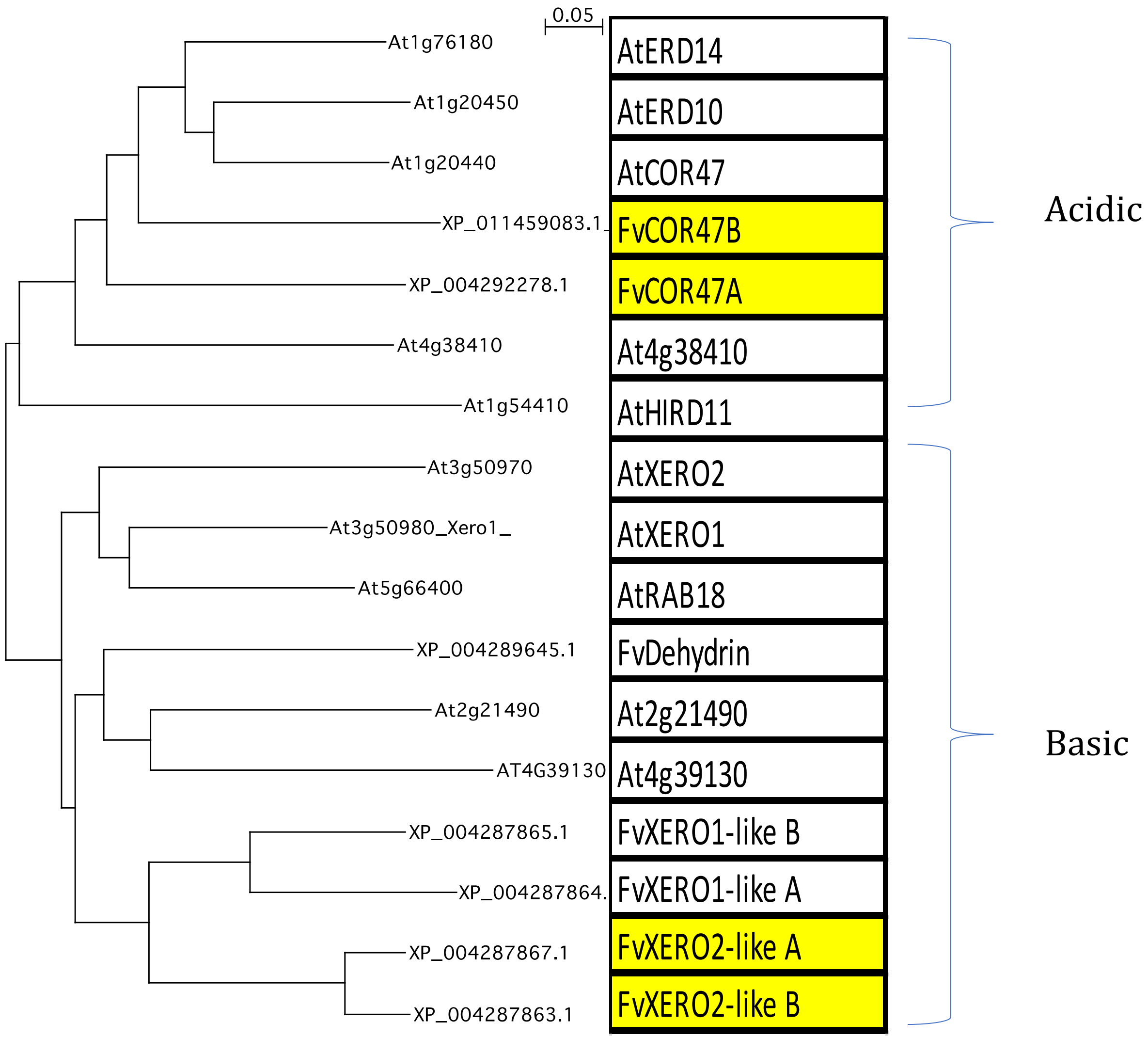

Figure S3: Phylogenetic analysis showing the relationship between ten *A. thaliana* dehydrins and the seven putative *F. vesca* dehydrin proteins identified in this study. Shaded boxes (yellow) indicate those examined in this study. Column to the right shows accepted common names for these genes.

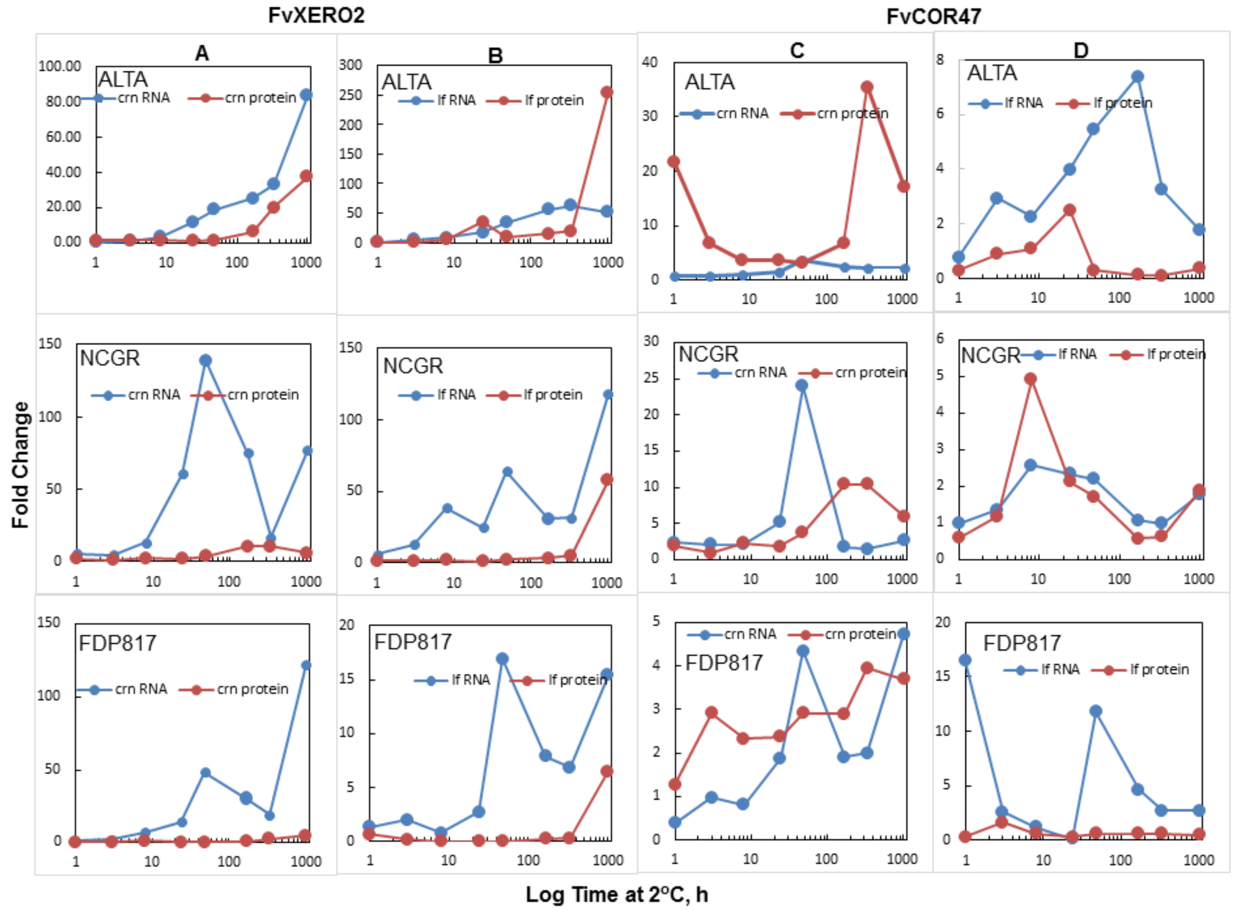

Figure S4: Corresponding changes of FvXERO2 and FvCOR47 transcript and protein in leaves and crowns of three *F. vesca* genotypes in response to LT. Changes in *FvXERO2* and *FvCOR47* transcript levels (Blue line, data from Figure 3) and FvXERO2 and FvCOR47 protein (orange line, data from Figure 5) in response to cold (2°C). For visualization purposes the standard deviations (see Figure 3 and Figure 5) have been removed. Panel **A** and **C** (left) are from crown tissues while Panel **B** and **D** (right) are from leaf tissues.

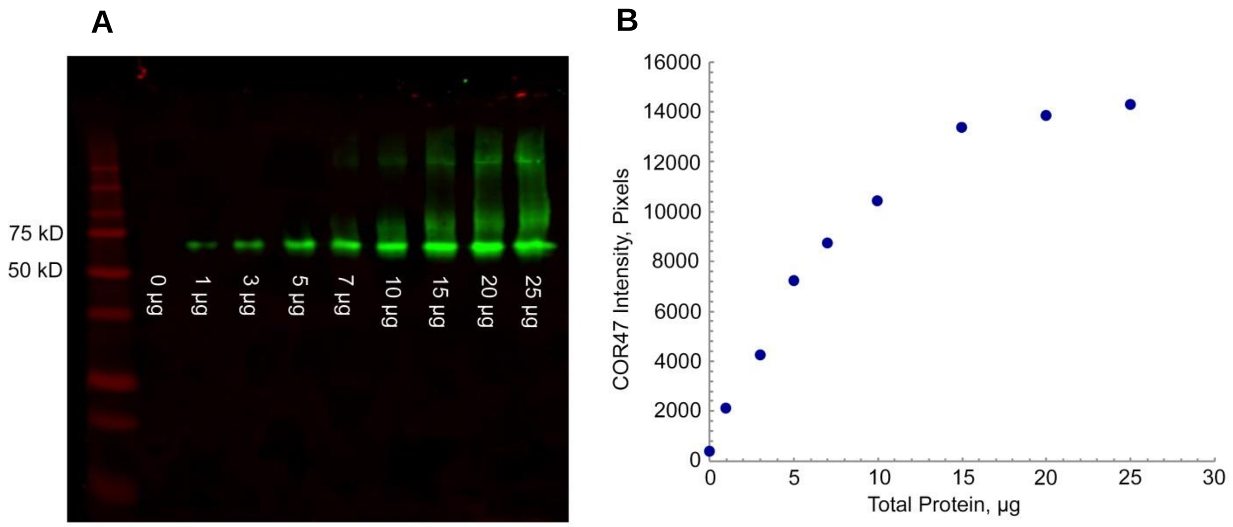

Figure S5: Quantitation of FvCOR47 is linear over a 10-fold range of protein (maximum of 10 µg). From 1 to 25 µg total protein extracted from cold-treated crowns (42 d) of the genotype 'ALTA' was loaded on 12% SDS-PAGE gel and blotted with antibody raised against COR47. Panel **A**, western blot, Panel **B**, Quantitation of gel shown in panel **A**. Since this (42 d of cold in 'ALTA' crowns) represents the highest levels of COR47 response, gels were generally loaded with 1-10 µg of total extract protein.

Table S1: (A) Alignment of the CBF candidates; their AP2 domain and immediately flanking regions are compared to the CBF consensus (JAGLO*)* [1]. Note only XP_004298771.1 (FvCBF1E) and XP_004308117.1 (FvCBF1B.1) contain the CBF consensus C-terminal DSAWR sequence and contain near identity at the N-terminal end of the AP2 domain. The double line above the sequences indicates the AP2 domain based upon [2]. The single lines indicate CBF consensus regions immediately flanking that domain. The alignment was performed with M-COFFEE (www. tcoffee.crg.cat) [3] which uses multiple alignment tools to create a consensus alignment with the color indicating good (red), average (tallow), poor (green) and bad (blue) agreement between the different methods.

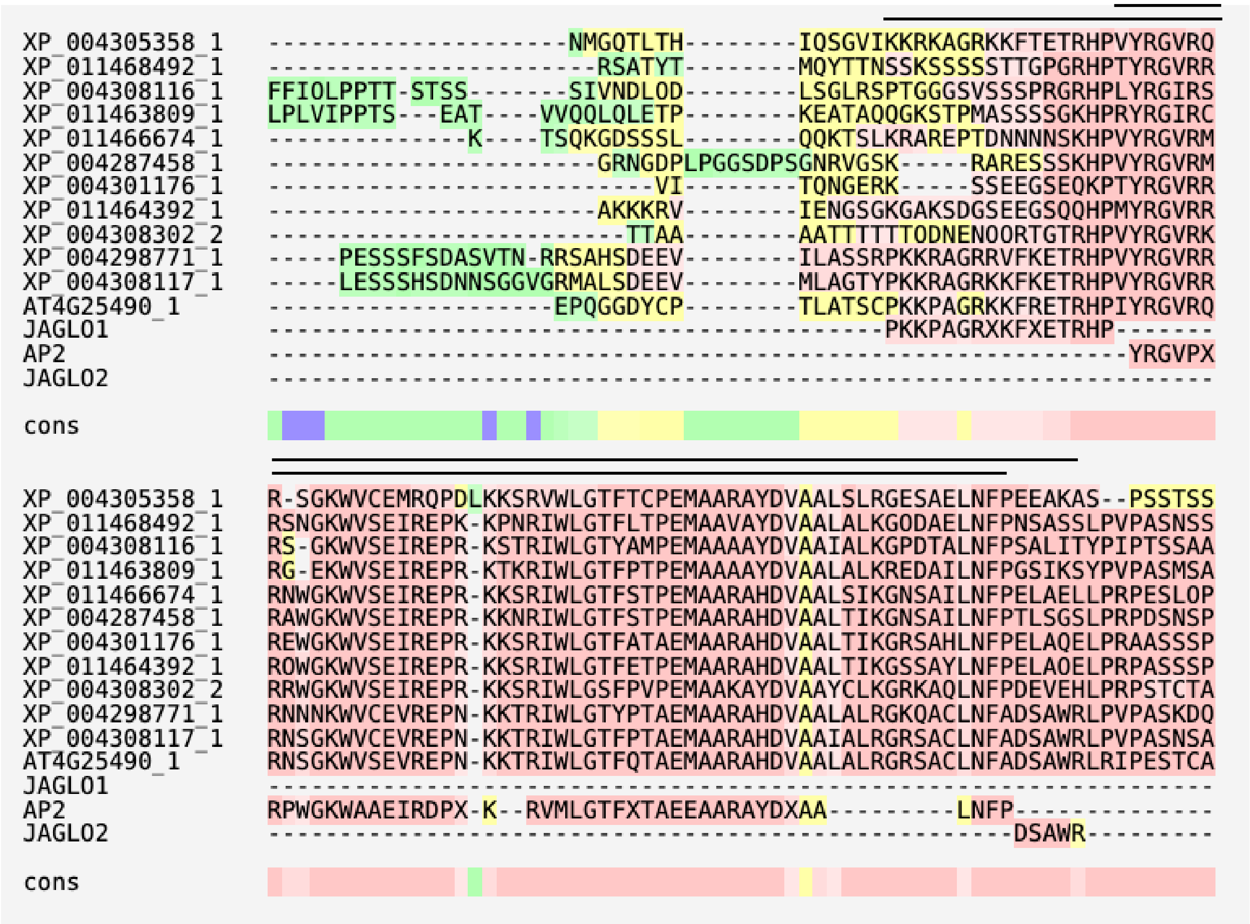

(B) Amino acid sequences used for phylogenetic analysis of CBFs.

>XP_004298771.1 PREDICTED: dehydration-responsive element-binding protein 1E [Fragaria vesca subsp. vesca]
MNSSTSQQQLSDSNLQQQPLDSSSSIDKPESSSFSDASVTNRRSAHSDEEVILASSRPKKRAGRRVFKET
RHPVYRGVRRRNNNKWVCEVREPNKKTRIWLGTYPTAEMAARAHDVAALALRGKQACLNFADSAWRLPVP
ASKDQLDIRRAAAEAAETFRPQEFGGVSSSCEDDYYQESNNEADQEVAKISLDSAEKKNEGFYEDEEAVF
DMPRLLTSMAEGLLLSPPRHYMDSINWGDADDVESEPNFSLWSYSV
>XP_004305358.1 PREDICTED: dehydration-responsive element-binding protein 1B-like [Fragaria vesca subsp. vesca]
MLSDASAASLMAYREEPAAAAALSSPASVLMNMGQTLTHIQSGVIKKRKAGRKKFTETRHPVYRGVRQRS
GKWVCEMRQPDLKKSRVWLGTFTCPEMAARAYDVAALSLRGESAELNFPEEAKASPSSTSSMSLLELTSM
VNLENVEVESRSSRIGYVDEEELFNMPELLDSLAEGLILTPPAMQKGFNWDDYDLENAVEFSLWSDD
>XP_011468492.1 PREDICTED: ethylene-responsive transcription factor ERF024 [Fragaria vesca subsp. vesca]
MACMPHRVCHRSATYTMQYTTNSSKSSSSSTTGPGRHPTYRGVRRRSNGKWVSEIREPKKPNRIWLGTFL
TPEMAAVAYDVAALALKGQDAELNFPNSASSLPVPASNSSRDIQAAAASAAAAISAAAAAMGVGNLATSS
WPSTESSHGHMLNDHEFVDEDLIFDMPNVLMNMAEGMLLSPPRLDIAGDHDALATEEHGTLWKFN
>XP_004308116.1 PREDICTED: ethylene-responsive transcription factor ERF027 [Fragaria vesca subsp. vesca]
MADPYGNVPKSDEQPSDSNPPFFIQLPPTTSTSSSIVNDLQDLSGLRSPTGGGSVSSSPRGRHPLYRGIR
SRSGKWVSEIREPRKSTRIWLGTYAMPEMAAAAYDVAAIALKGPDTALNFPSALITYPIPTSSAASDIRA
AAARAAASRAEVIRQGESSSAGGVENVDRSGGGRGRRAAVTSDDGGGSQEFMDEEALLNMPNLLADMAEG
MLVSPPRGPANDSPENSDGESLWSY
>XP_011463809.1 PREDICTED: ethylene-responsive transcription factor ERF027-like [Fragaria vesca subsp. vesca]
MADPNTPANNLRQMIEDQPAPTQLITLDFPPPPPSPTLPQPPQPPPPPPSHSLALLPLVIPPTSEATVVQ
QLQLETPKEATAQQGKSTPMASSSSGKHPRYRGIRCRGEKWVSEIREPRKTKRIWLGTFPTPEMAAAAYD
VAALALKREDAILNFPGSIKSYPVPASMSALDIRAAATAAATAAAAETTLKTSGSRESTPSSVGQQQKEY
DDMLSINSSALASSVGADFMDEEEIFGMPNLLVDMAEGMLVSPPRINSPPSDYDSPEYSDGGESLWNYD
>XP_011466674.1 PREDICTED: ethylene-responsive transcription factor TINY [Fragaria vesca subsp. vesca]
MAAEPNNSEAESTTTSNSSSLCSVASSPCLSGLLQTATHPKSPSNTTKNPKTSQKGDSSSLQQKTSLKRA
REPTDNNNNSKHPVYRGVRMRNWGKWVSEIREPRKKSRIWLGTFSTPEMAARAHDVAALSIKGNSAILNF
PELAELLPRPESLQPPDIQAAAAKAAAMVHLDSTTTTSSSSTSSSSSSSEGLESGEDELGEIVELPNIED
SFNSGESRSEFILVNDTVDIGWLYDHPPLMGLDHFEEDLVSWDIQMIT
>XP_004287458.1 PREDICTED: ethylene-responsive transcription factor TINY [Fragaria vesca subsp. vesca]
MTKDPTRSDTTTESTSFNSSSFSSSPCSGRNGDPLPGGSDPSGNRVGSKRARESSSKHPVYRGVRMRAWG
KWVSEIREPRKKNRIWLGTFSTPEMAARAHDVAALTIKGNSAILNFPTLSGSLPRPDSNSPRDIQAAAAK
AASMENLDPPPPQPQPPSSALSQSSSSSSSLEVTDAGTPEELGEIVELPRLGTSYESAESGSDFVFEDSV
EGWLYPPAQPWWQNGEFGEDYGYSRNFNEMLMAEGGVEDLLWQH
>XP_004301176.1 PREDICTED: ethylene-responsive transcription factor ERF039-like [Fragaria vesca subsp. vesca]
MESVITQNGERKSSEEGSEQKPTYRGVRRREWGKWVSEIREPRKKSRIWLGTFATAEMAARAHDVAALTI
KGRSAHLNFPELAQELPRAASSSPKDIQAAAAKAAALSYPSCSSTVSSSSSSSSSHEAQEHYAEPAQDSS
PSETINDDTFFDLPDLLLDVGHYRSDDAYCFSIVDTSISLEEPFLWD
>XP_011464392.1 PREDICTED: ethylene-responsive transcription factor ERF039-like [Fragaria vesca subsp. vesca]
MEEEQSTTSSSSSGSTTSAECEAGKKCKGAKKKRVIENGSGKGAKSDGSEEGSQQHPMYRGVRRRQWGKW
VSEIREPRKKSRIWLGTFETPEMAARAHDVAALTIKGSSAYLNFPELAQELPRPASSSPKDIQAAAAKAA
ALSYPNCRNSHETHEPEEARPCRDSSSTSPLSDEHNDDTFYDLPDLLIDVGQYHGDHEYYFPVAATWQLD
PMDTILRMEESFLWD
>XP_004308117.1 PREDICTED: dehydration-responsive element-binding protein 1B-like [Fragaria vesca subsp. vesca]
MDGGFSALSQYYDHNPVGSDLLESSSHSDNNSGGVGRMALSDEEVMLAGTYPKKRAGRKKFKETRHPVYR
GVRRRNSGKWVCEVREPNKKTRIWLGTFPTAEMAARAHDVAAIALRGRSACLNFADSAWRLPVPASNSAK

DIQTAAAEAAEAFRPNGEESEVVSKAAEAVEAESEQPVFYMDVEEDVFGMPGLLANMAEGMLLPPPQYSG
YGGDDMEGYADVSLWSYSI
>XP_004308302.2 PREDICTED: ethylene-responsive transcription factor ERF023
[Fragaria vesca subsp. vesca]
MYMEQPPFTEDDLITPTTTAAAATTTTTTQDNENQQRTGTRHPVYRGVRKRRWGKWVSEIREPRKKSRIW
LGSFPVPEMAAKAYDVAAYCLKGRKAQLNFPDEVEHLPRPSTCTARDIQAAAAKAAQTMKSSSTASRSTD
DQDHHRDVSDDGSADDFWGEIELPELMNSGTCYSNSCGWSTFSGEAAALTWPEGEACL
>AT4G25490.1 AtCBF1
MNSFSAFSEMFGSDYEPQGGDYCPTLATSCPKKPAGRKKFRETRHPIYRGVRQRNSGKWVSEVREPNKKTRIWLGTF
QTAEMAARAHDVAALALRGRSACLNFADSAWRLRIPESTCAKDIQKAAAEAALAFQDETCDTTTTNHGLDMEETMVE
AIYTPEQSEGAFYMDEETMFGMPTLLDNMAEGMLLPPPSVQWNHNYDGEGDGDVSLWSY
>**AT4G25470.1** AtCBF2
MNSFSAFSEMFGSDYESPVSSGGDYSPKLATSCPKKPAGRKKFRETRHPIYRGVRQRNSGKWVCELREPNKKTRIWL
GTFQTAEMAARAHDVAAIALRGRSACLNFADSAWRLRIPESTCAKEIQKAAAEAALNFQDEMCHMTTDAHGLDMEET
LVEAIYTPEQSQDAFYMDEEAMLGMSSLLDNMAEGMLLPSPSVQWNYNFDVEGDDDVSLWSY
>**AT4G25480.1** AtCBF3
MNSFSAFSEMFGSDYESSVSSGGDYIPTLASSCPKKPAGRKKFRETRHPIYRGVRRRNSGKWVCEVREPNKKTRIWL
GTFQTAEMAARAHDVAALALRGRSACLNFADSAWRLRIPESTCAKDIQKAAAEAALAFQDEMCDATTDHGFDMEETL
VEAIYTAEQSENAFYMHDEAMFEMPSLLANMAEGMLLPLPSVQWNHNHEVDGDDDDVSLWSY
>**AT5G51990.1** AtCBF4
MNPFYSTFPDSFLSISDHRSPVSDSSECSPKLASSCPKKRAGRKKFRETRHPIYRGVRQRNSGKWVCEVREPNKKSR
IWLGTFPTVEMAARAHDVAALALRGRSACLNFADSAWRLRIPETTCPKEIQKAASEAAMAFQNETTTEGSKTAAEAE
EAAGEGVREGERRAEEQNGGVFYMDDEALLGMPNFFENMAEGMLLPPPEVGWNHNDFDGVGDVSLWSFDE
>NP_001234123.1 SLCBF1
MNIFETYYSDSLILTESSSSSSSSFSEEEVILASNNPKKPAGRKKFRETRHPIYRGIRKRNSGKWVCEVREPNKKT
RIWLGTFPTAEMAARAHDVAALALRGRSACLNFSDSAWRLPIPASSNSKDIQKAAAQAVEIFRSEEVSGESPETSEN
VQESSDFVDEEAIFFMPGLLANMAEGLMLPPPQCAEMGDHCVETDAYMITLWNYSI
>AAS77821.1 SLCBF2
MDIFESYYSNSFVESLLSSSLSISDTNNLNHYSPNEEVIILASNNPKKPAGRKKFRETRHPVYRGIRKRNSGKWVCE
VREPNKKTRIWLGTFPTAEMAARAHDVAAIALRGRSACLNFADSVWRLPIPASSNSKDIQKAAAEAAEIFRSEEVSG
ESPETSENVQESSDFVDEEALFSMPGLLANMAEGLMLPPPQCLEIGDHYVELADVHAYMPLWNYSI
>AY497899.1 SLCBF3
MFYSDPRIESCSSFSDSIRANHSDEEVILASNNPKKPAGRKKFRETRHPVYRGVRKRNSGKWVCEVREPNKKTRIWL
GTFPTAEMAARAHDVAAIALRGRSACLNFADSAWRLPTPDSSDTKDIQKAAAQAAEIFRPLKSEEEESVVKDQSTTP
DDMFFMDEEALFCMPGLLTNMAEGLMVPPPQCTEMGDHVEADDMPLWSYSI
>Glyma09g27180.1 GmDREB1A;1
MFTLNHSSDLYHVSPELSSSLDTSSPASEGSRGVAFSDEEVRLAVRHPKKRAGRKKFRETRHPVYRGVRRRNSDKWV
CEVREPNKKTRIWLGTFPTPEMAARAHDVAAMALRGRYACLNFADSAWRLPVPATAEAKDIQKAAAEAAQAFRPDQT
LKNANTRQECVEAVAVAVAETTTATAQGVFYMEEEEQVLDMPELLRNMVLMSPTHCLGYEYEDADLDAQDAEVSLWN
FSI
>Glyma16g32330.1 GmDREB1B;1
MYTLNHSSYLYHVSPELSSSLDSSSPASEGSRGVAFSDEEVRLAVRHPKKRAGRKKFRETRHPVYRGVRRRNTDKWV
SEVREPNKKTRIWLGTFPTPEMAARAHDVAAMALRGRYACLNFADSTWRLPIPATANAKDIQKAAAEAAEAFRPSQT
LENTNTKQECVKVVTTTTITEQKRGMFYTEEEEQVLDMPELLRNMVLMSPTHCIGYEYEDADLDAQDAEVSLWSFSI
>Glyma20g29410.1 GmDREB1B;1
MFSINHFSDPDATSPGSGGSSRPALSDEDFFLAASNPKKRAGRKKFRETRHPVYRGVRRRDSGKWVCEVREPNKKSR
IWLGTFPTAEMAARAHDVAAIALRGRSACLNFADSASRLPVPATAEARDIQKAAAEAAEAFRPGKDDDAVAERVAAT
ATEREEEQEEGMVPEYLRNMVLMSPTHCFGSDEYGSADVEFDDAEVSLWSYSI
> Glyma10g38440.1 GmDREB1B;2
MFSINHFSAPDANSPGSGSGTSRPVPALSDEDYFLAASNPKKRAGRKKFRETRHPVYRGVRRRDSGKWVCEVREPNK
KSRIWLGTFPTAEMAARAHDVAALALRGRSACLNFADSASRLPVPATAEARDIQKAAAEAAEAFRPGKDDGAVVATA
TEREEEEEKVVPEYLRNMVLMSPTHCFGSDEYGSADVEFDDAEVRLWSYSI
>AT5G05410.1, ATDREB2A
MAVYDQSGDRNRTQIDTSRKRKSRSRGDGTTVAERLKRWKEYNETVEEVSTKKRKVPAKGSKKGCMKGKGGPENSRC
SFRGVRQRIWGKWVAEIREPNRGSRLWLGTFPTAQEAASAYDEAAKAMYGPLARLNFPRSDASEVTSTSSQSEVCTV
ETPGCVHVKTEDPDCESKPFSGGVEPMYCLENGAEEMKRGVKADKHWLSEFEHNYWSDILKEKEKQKEQGIVETCQQ

QQQDSLSVADYGWPNDVDQSHLDSSDMFDVDELLRDLNGDDVFAGLNQDRYPGNSVANGSYRPESQQSGFDPLQSLN
YGIPPFQLEGKDGNGFFDDLSYLDLEN
>AT5G11590.1 ATTINY2 ATDREB subfamily A-4
maeeyyslrservtqllvpnsesdsvsdkskaeqsekktkrgrdsgkhpvyrgvrmrnwgkwvseireprkksriwl
gtfptpemaarahdvaalsikgtaailnfpeladsfprpvslsprdiqtaalkaahmepttsfssstsssslssts
sleslvvmdlsrteseelgeivelpslgasydvdsanlgnefvfydsvdyclypppwgqssednyghgispnfghgl
swdl

(C) Amino acid sequences used for phylogenetic analysis of Dehydrins. Locations of the "K" (red) , the "S" (blue) and "Y" (green) sequences are shown for the *Fragaria vesca* sequences).

>At1g20440 (COR47)
MAEEYKNNVPEHETPTVATEESPATTTEVTDRGLFDFLGKKEEEVKPQETTTLESEFDHKAQISEPELAA
EHEEVKENKITLLEELQEKTEEDEENKPSVIEKLHRSNSSSSSSSDEEGEEKKEKKKKIVEGEEDKKGLV
EKIKEKLPGHHDKTAEDDVPVSTTIPVPVSESVVEHDHPEEEKKGLVEKIKEKLPGHHDEKAEDSPAVTS
TPLVVTEHPVEPTTELPVEHPEEKKGILEKIKEKLPGYHAKTTEEEVKKEKESDD
>At1g76180 (ERD14)
MAEEIKNVPEQEVPKVATEESSAEVTDRGLFDFLGKKKDETKPEETPIASEFEQKVHISEPEPEVKHESL
LEKLHRSDSSSSSSSEEEGSDGEKRKKKKEKKKPTTEVEVKEEEKKGFMEKLKEKLPGHKKPEDGSAVAA
APVVVPPPVEEAHPVEKKGILEKIKEKLPGYHPKTTVEEEKKDKE
>At4g38410 (Q9SVE4)
MADHPRSTEQQEADAAASKGCGMFDFLKKKPEDVHSSENARVTKEPKEEEKPSLAERFHLSDSSSSDEEA
GENGEKKEKKKKKKKNEVAEDQCETEEKIPAGIGHEDGKEKGFMEKIKDKLPGGHNGKPEAEPHNDKAKE
KGFMEKIKEKLPGHTNDEKKKET
>At2g21490 (X91920)
MADLRDEKGNPIHLTDTQGNPIVDLTDEHGNPMYLTGVVSSTPQHKESTTSDIAEHPTSTVGETHPAAAP
AGAGAATAATATGVSAGTGATTTGQQHHGSLEEHLRRSGSSSSSSSEDDGQGGRRKKSIKEKIKEKFGSG
KHKDEQTPATATTTGPATTDQPHEKKGILEKIKDKLPGHHNHNHP
>At3g50970 (Xero2)
MNSHQNQTGVQKKGITEKIMEKLPGHHGPTNTGVVHHEKKGMTEKVMEQLPGHHGATGTGGVHHEKKGMT
EKVMEQLPGHHGSHQTGTNTTYGTTNTGGVHHEKKSVTEKVMEKLPGHHGSHQTGTNTAYGTNTNVVHHE
KKGIAEKIKEQLPGHHGTHKTGTTTSYGNTGVVHHENKSTMDKIKEKLPGGHH
>At1g20450 (ERD10)
MAEEYKNTVPEQETPKVATEESSAPEIKERGMFDFLKKKEEVKPQETTTLASEFEHKTQISEPESFVAKH
EEEEHKPTLLEQLHQKHEEEEENKPSLLDKLHRSNSSSSSSSDEEGEDGEKKKKEKKKKIVEGDHVKTVE
EENQGVMDRIKEKFPLGEKPGGDDVPVVTTMPAPHSVEDHKPEEEEKKGFMDKIKEKLPGHSKKPEDSQV
VNTTPLVETATPIADIPEEKKGFMDKIKEKLPGYHAKTTGEEEKKEKVSD
>AT4G39130 (Q9T022)
MADLKDERGNPIYLTDAHGEPAQLMDEFGNAMHLTGVATTVPHLKESSYTGPHPITAPVTTTNTPHHAQP
ISVSHDPLQDHDLRWFGTSSTEENGEGVGRKTNITDETKSKLGVDKPSAATVTGSGSGSVHEKKGFFKKI
KEKLSGHHNDL
>At5g66400 (Rab18)
MASYQNRPGGQATDEYGNPIQQQYDEYGNPMGGGGYGTGGGGGATGGQGYGTGGQGYGSGGQGYGTGGQG
YGTGTGTEGFGTGGGARHHGQEQLHKESGGGLGGMLHRSGSGSSSSSEDDGQGGRRKKGITQKIKEKLPG
HHDQCGQAQAMGGMGSGYDAGGYGGEHHEKKGMMDKIKEKLPGGGR
>At3g50980(Xero1)
MESYQNQSGAQQTHQQLDQFGNPFPATTGAYGTAGGAPAVAEGGGLSGMLHRSGSSSSSSSEDDGLGGRR
RKKKGITEKIKEKLPGHHDSNKTSSLGSTTTAYDTGTVHHEKKGMMEKIKEKLPGGHH
>At1g54410

MAGLINKIGDALHIGGGNKEGEHKKEEEHKKHVDEHKSGEHKEGIVDKIKDKIHGGEGKSHDGEGKSHDG
EKKKKKDKKEKKHHDDGHHSSSSDSDSD
> XP_004287864.1
MAHQSENGRDTTADESGNPIRRTEDPVHHQGVTEATTGNGTHDTITGYGTHETTGTGHKTHGTTGTGNGT
HETTETGHKTHGTTGTGNGTHGRAARATGPTGFAATTAGGQGLLHHRSDGSSSSSEDDGYGGRRKKEEKE
KEGLEEKIKEKLPGGNSDHSGTNNTPPSDTIPHEKKGMMDKIKEKLPGHHEIDHSSTIDICT
> XP_004287865.1
MAHFQSEYDRDKTTDEFGNPIRRTEDPVHHGVTGATAGYGSHDTTGTGYGTHGTTGTDYGTHGTTGTGFG
THGTGPTGFAATTAGGVTSVPLHRSDSSSSSEDDGYGGRRKKKGLKEKIKEKLPGGKHRSDDPYGATTTP
YGGGQHQEKGMMDKIKEKLPGHH
> XP_004287863.1
MAQYQSGYPTQSPTDEYGNPVGRKDEYGNPIHTGATTGQGVHPGTGTGHGGVTTLTRSGSGSSSSEDDGC
GGRRKKGMKEKIKEKLPGGTHKTDDPYGTAPTTAPYGGQQQDRGVMDQIKQKIPGGQKQDPYSTGQHTTT
PGYGVAGEHHEKKGMVDQIKEKIPGGQKQDPYSTAHHTGATPGYGVAGEHHEKKGMVDQVKEKLPGGLTG
GQDPHNTTHTHTTAAPAYGTGTGEHQEKKGMMDKIKEKLPGGHTTTR
>XP_004292278.1
MADQYQKETKENHAAAKVEEEPATGCGMFDFLKKKENEKPQPEEHKHTTAEKLHHHDGNDSSSSSSDEEG
GEKKKKGLKGKIKEKIASKKNEEDHHSPIPAAKSNDIESGVSNGQEKKGFIEKIKEKLPGQHKEAEAGAP
PEYHSPHEGEPKEKKGIMEKIKEKLPGGNKNEEEKPKEY
>XP_004289645.1
MSEIRDEFGNPVPLTDQYGNPVQLTDELGRPIHLSGIATTEGAQGQAIRTGEHHTGTQGSTGTGLHIPGT
GTHAPGTGVHVPGTGQHVPGTGAHIPGTGIHVPGTGEHVPGTGAHVPGTGAHVPGTGAHMPGMTAGDGVG
LMAAVHVTDKPKTVGEHLHQERHTGVAGEHRRSSSSSSSEEDDGKGGRIRKKKGLKEKIKETFGGKHKDE
HPQAGPTFKTPTTTPQEHEKKGMMEKIKEKLPGHHAH
> XP_004287867.1
MAQYQSGYPTQSPTDEYGNPVGRKDEYGNPIHTGATTGQGVHPVTGTGTGTGHGGVTTLTRSGSSSSSSE
DDGCGGRRKKGMKEKIKEKLPGGTRKTDDPYGTAPTTAPYGGQNQDRGVMDKIQEKIPGGQKQDPYSTGQ
HTSATPGYGVAGEHEKKGMVDQVKEKLPGGLTGGQDPHNTTHTHTTAAPAYGTGTGEQQEKKGMVDQVKE
KLPGGLTGGQDPHNTTHTHTTAAPAYGTGTGEQQEKKGMMDKIKEKLPAGTTR
>XP_011459083.1)
MAGEYNKGHEYETKAEEGGPVETQDRGMFDFLGKKKEEEKPVHHEQMVSEFEKVKVSEPEPAYVDCSSKP
VEHGYHHEEPKEEHKKEEEKKHETLSEKLRRSDSSSSSSSDEEGDDREKKKRKEKKGLKEKLKEKIAGDK
EEEEKKKHGYEQDTEVPVEKFHEEQEHPYDHGPHHHEEPKVEPTVAYTEEQKKEDEKKGFLEKIKDKLPG
HKKPEDVPVASPPPPEYENVEPAYHEGEVKEKKGLLEKIKEKIPGYHPKTEEEKLEKEKEKPTGSY

Table S3: Summary of threshold Cts for all targets under non-acclimating conditions (0 h in the cold). Summary of average threshold Cts at steady state (prior to cold treatments, i.e., 0 time). These are useful for comparison of constitutive (basal) levels in transcripts in the three genotypes. Panel **A**. is raw Cts, Panel **B** is normalized to PP2A levels in ALTA (0 time in leaves); to provide a direct comparison to levels in the crowns. In general, very comparable levels of transcript appear to be present in all accessions. However, in ALTA crowns (AL0HR1), GEM, CBFs, XERO2, and COR47 qRT-PCR products are significantly higher than the other (less cold tolerant) accessions (RED), NCGR1363 (NL0HR1) and FDP819 (FL0HR1) (**BOLD**).

		LEAF			CROWN		
**A**							
**RAW Ct Values**							
		AL0HR1	NL0HR1	FL0HR1	AL0HR1	NL0HR1	FL0HR1
	AVG Cts						
	PP2A	25.605	26.351	25.774	23.822	22.382	24.889
	GEM	26.417	27.688	26.39	22.801	24.288	26.047
	BTB	28.095	29.308	28.93	29.326	27.613	27.901
	P2C16	27.782	28.062	27.992	25.037	24.905	25.499
	P2C27	26.309	27.212	26.728	26.821	26.55	28.044
	RHA11	30.508	30.004	30.02	27.376	28.504	28.782
	CBF1B.1	35.203	34.972	33.525	22.629	32.861	32.96
	CBF1E	33.013	32.394	31.167	28.593	31.188	31.518
	XERO2	24.873	25.484	24.584	21.351	22.904	23.915
	COR47	21.282	22.677	22.579	19.962	20.776	22.704
	ADH	30.388	30.796	28.447	24.69	22.51	22.93
**B**							
**Normalized to PP2A, in AL0HR1, LEAF**							
		AL0HR1	NL0HR1	FL0HR1	AL0HR1	NL0HR1	FL0HR1
	AVG Cts						
	PP2A	25.605	25.605	25.605	25.605	25.605	25.605
	GEM	26.417	26.904	26.217	24.508	27.785	26.796
	BTB	28.095	28.478	28.740	31.521	31.589	28.704
	P2C16	27.782	27.268	27.808	26.911	28.491	26.233
	P2C27	26.309	26.442	26.553	28.828	30.373	28.851
	RHA11	30.508	29.155	29.823	29.425	32.609	29.610
	CBF1B.1	35.203	33.982	33.305	**24.323**	**37.593**	**33.908**
	CBF1E	33.013	31.477	30.963	**30.733**	**35.679**	**32.425**
	XERO2	24.873	24.763	24.423	**22.949**	**26.202**	**24.603**
	COR47	21.282	22.035	22.431	**21.456**	**23.768**	**23.357**
	ADH	30.388	29.924	28.260	26.538	25.751	23.590

Table S4: qRT-PCR primers used in this study.

Gene Name	Gene ID (NCBI)	Sequence (5'-3')
FvADH	101311341	R:GGTTTTGTCCCTTGGCTT
		F:GCACCTCCTCAGGCTAA
FvBTB	101306347	R:ACTATTTGACACTGCACTTCT
		F:CCTTTCATCTTCACAAGTTCCC
FvCBF1D	101302987	R:CGCTGTTGTTATCGGAATGAGA
		F:CAGTAGTAATGGATGGTGGT
FvCBF1F	101298019	R:CTGGCGTCGGAGAAGGA
		F:AGCTCCACTTCCCAGCA
FvCOR47, A&B	101303769	R:TCTCGAACTCGCTGACC
		F:AGTACAACAAAGGCCACGA
FvGEM	101299886	R:CTGGCGCAACCTTAAAAC
		F:GGAGAGATAGCAGGCGAA
FvRHA11	101302171	R:GCGACGTCTTCCGGTTC
		F:TGTCTTCCACCCGGACT
FvXERO2, A & B	101314123	R:GCCTCCGTGACCAGTTC
		F:TACCCCACTCAGTCTCCC
FvPP2A	101300814	R:CTTCTCCACAACCGACT
		F:AGGACAGAGTACCCAACA

COR47 Isogene specific primers

COR47 A	101300217	R: TGCTGGCGATCTTCTCCTTG
		F: GCTCGTCTAGCAGTGACGAA
COR47 B	101303769	R: CGTCACTTGACGAGCTGGAG
		F: GCAAACCTGTTGAGCATGGC

COR47 B INT	101303769	R: CCTGCTCGTAGCCGTGT
		F: TGGCTATCATCACGAGGAACC

Reference

1. Jaglo, K. R.; Kleff, S.; Amundsen, K. L.; Zhang, X.; Haake, V.; Zhang, J. Z.; Deits, T.; Thomashow, M. F., Components of the Arabidopsis C-repeat/dehydration-responsive element binding factor cold-response pathway are conserved inbrassica napus and other plant species. *Plant physiology* **2001,** 127, (3), 910-917.
2. Riechmann, J. L.; Meyerowitz, E. M., The AP2/EREBP family of plant transcription factors. *Biological chemistry* **1998,** 379, 633-646.
3. Notredame, C.; Higgins, D. G.; Heringa, J., T-Coffee: A novel method for fast and accurate multiple sequence alignment. *Journal of molecular biology* **2000,** 302, (1), 205-217.
